# Digging into Understudied Sediments: What *Lumbriculus variegatus* Reveals about Fluoxetine Exposure from the Cellular to the Individual Level

**DOI:** 10.1021/acs.est.5c12822

**Published:** 2026-09-03

**Authors:** Jacqueline Hilgendorf, Vânia Calisto, Diana Campos, Diogo N. Cardoso, Joana Fernandes, Diana L.D. Lima, Jesper G. Sørensen, Érika M.L. Sousa, Susana Loureiro

**Affiliations:** † CESAM-Centre for Environmental and Marine Studies & Department of Biology, 56062 University of Aveiro, Campus Universitário de Santiago, Aveiro 3810-193, Portugal; ‡ CESAM-Centre for Environmental and Marine Studies & Department of Chemistry, 56062 University of Aveiro, Campus Universitário de Santiago, Aveiro 3810-193, Portugal; § H&TRC - Health & Technology Research Center, Coimbra Health School, 196356 Polytechnic University of Coimbra, Rua 5 de Outubro, Coimbra 3045-043, Portugal; ∥ Department of Biology, Aarhus University, Ny Munkegade 114, Building 1540, Aarhus C DK-8000, Denmark

**Keywords:** pharmaceuticals, sediment ecotoxicology, behavior, oxidative stress, energy metabolism

## Abstract

Sediments accumulate persistent and toxic compounds, yet assessing their effects on benthic species remains challenging. Especially antidepressants, such as fluoxetine, are of concern due to their sediment affinity and potential to disrupt neuroendocrine functions at low concentrations. This study investigated the chronic effects of fluoxetine in the sediment-dwelling worm *Lumbriculus variegatus* through two 28-day sediment exposures (0.00025–25 mg/kg) conducted in accordance with OECD 225, with modifications. Endpoints encompassed multiple biological levels, including biochemical markers of neurotoxicity (cholinesterase), oxidative stress (catalase, glutathione-*S*-transferase, total glutathione), damage (lipid peroxidation), energy metabolism (lipids, sugars, proteins, electron transport system), physiological responses (oxygen consumption), three novel behavioral assays (chemical avoidance, feeding, phototaxis), and conventional apical endpoints (mortality, growth, reproduction). Effects on the non-conventional endpoints occurred at environmentally relevant sediment concentrations. Additionally, oxidative stress and behavioral responses were substantially more sensitive endpoints of fluoxetine exposure than the conventional endpoints growth, reproduction, and mortality. Integrating responses across biological levels, we propose a qualitative Adverse Outcome Pathway linking molecular and cellular events to individual-level effects. Our findings demonstrate that environmentally relevant fluoxetine concentrations elicit ecologically meaningful effects in *L. variegatus* that current regulatory frameworks would likely overlook, emphasizing the need for standardized behavioral assays in sediment risk assessment.

## Graphical abstract

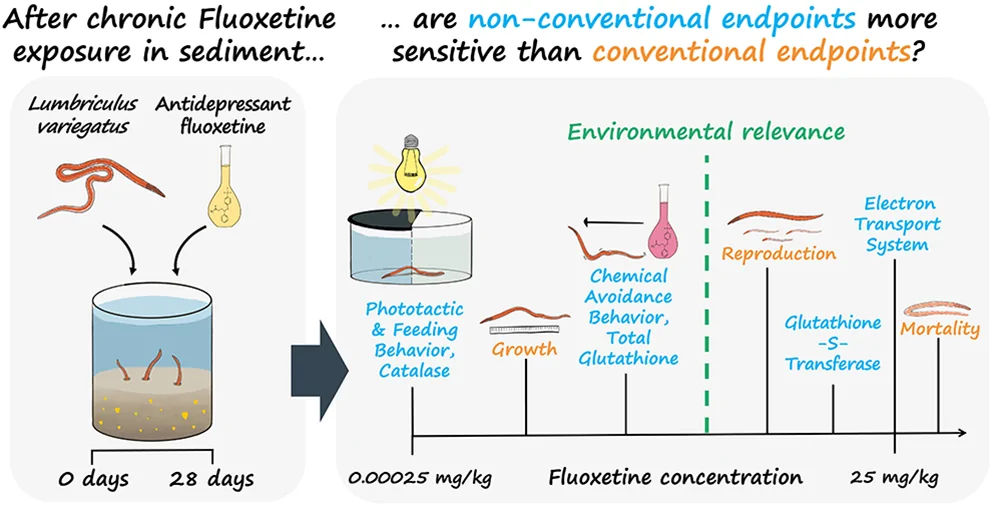

## 1 Introduction

Sediments act as sinks for persistent, bioaccumulative, and toxic compounds, yet assessing their effects on benthic organisms remains challenging.^1^ Pharmaceuticals discharged through wastewater treatment plants pose a particular threat, as many adsorb strongly to sediments.^2^ Designed to act at low concentrations, pharmaceuticals can disrupt the hormonal or nervous systems, causing adverse effects in aquatic organisms.^3^

Antidepressants are of particular concern, as their use has more than doubled in Europe between 2000 and 2019,^4^ leading to higher discharges.^5^ Many antidepressants, such as the serotonin reuptake inhibitors (SSRIs) fluoxetine or sertraline, accumulate in sediments,^6^ where they potentially have adverse effects on sediment-dwelling organisms. The mode of action (MoA) of SSRIs involves inhibiting the serotonin transporter and increasing synaptic serotonin levels. The monoamine serotonin (5-hydroxytryptamine, 5-HT) regulates key neurotransmitters such as glutamate, dopamine, and (acetyl)­choline. By modulating serotonin, antidepressants can have neuroendocrine effects, disrupting the hormonal system and altering physiological functions and behavior across a wide range of aquatic organisms.^7–9^ SSRI effects often follow non-monotonic dose–response curves (NMDRCs), where low-level effects may disappear or reverse at higher doses.^10^ Consequently, assessing their environmental impact is challenging, and critical effects may remain undetected when only conventional endpoints (i.e., those recommended by standardized guidelines), such as mortality, growth, and reproduction, are used.^11,12^

Understanding the effects of neuroendocrine chemicals (e.g., SSRIs) requires linking molecular and cellular responses to adverse outcomes at higher biological levels through the Adverse Outcome Pathway (AOP) framework.^13^ For years, scientists have advocated for the inclusion of AOPs in environmental risk assessment (ERA) practices,^14,15^ and the European Chemicals Agency (ECHA) has now recognized their importance in its KARC report, for addressing the emerging hazards, neurotoxicity, immunotoxicity, and metabolic toxicity.^16^ For these emerging hazards, conventional endpoints, such as mortality, growth, and reproduction, may not be sufficiently sensitive to detect toxic effects; therefore, non-conventional endpoints (NCEs; i.e., going beyond standardized guidelines) are needed.^17^ This study measured NCEs, such as biochemical endpoints related to neurotoxicity (cholinesterase (ChE)), oxidative damage (lipid peroxidation (LPO)), oxidative stress (catalase (CAT), glutathione-*S*-transferase (GST) activities, total glutathione (TG) content), and energy metabolism (protein, sugar, lipids, electron transport system (ETS)), as well as behavioral responses (chemical avoidance, feeding, phototaxis) alongside conventional endpoints (mortality, growth, reproduction) to evaluate SSRI effects across multiple biological levels and develop a qualitative AOP.

To evaluate the effects of SSRIs in sediments, the aquatic oligochaete *Lumbriculus variegatus* was selected as the model organism, as it is the only sediment-dwelling organism that is recommended by the OECD (Organization for Economic Co-operation and Development) guidelines and feeds mainly on sediment.^12,18^ *L. variegatus* is considered an ecological engineer that plays a key role as a bioturbator^19^ and serves as prey for fish and other organisms.^20^ Furthermore, *L. variegatus* possesses functional 5-HT receptors and serotonin,^21,22^ and its asexual reproduction via architomy (i.e., splitting into parts that then regenerate missing organs) is hypothesized to be regulated by epidermal serotonin-immunoreactive nerve rings,^23^ making it particularly relevant for SSRI exposure studies.

Fluoxetine was selected as the model SSRI due to its extensive use^24^ and high environmental discharges.^5^ It is considered lipophilic, has a high adsorption potential, and rapidly partitions from the water column into sediments.^25–27^ Consequently, fluoxetine and its metabolite norfluoxetine tend to reach higher concentrations in sediments and biota than in the aqueous phase.^28^ As a polyfluorinated pharmaceutical with highly stable C–F bonds, fluoxetine meets broad structural definitions of PFAS (per- and polyfluoroalkyl substances), also referred to as “forever chemicals”.^27^ Although it is not typically classified as a conventional PFAS, this stability raises concerns about environmental persistence.^27^ Environmental concentrations detected in river sediments range from 0.019 mg/kg in the US^28^ and 0.025 mg/kg in Spain^29^ to 0.17 mg/kg in Brazil.^30^ In laboratory studies, fluoxetine remained stable in sediment for 8 weeks after exposure application.^26^ In soil amended with sewage sludge, fluoxetine showed very low biodegradability over >200 days, indicating long persistence in solid phases.^31^

Regarding fluoxetine effects on *L. variegatus*, previous studies observed an LC50 (lethal concentration 50%) of 81.4 mg/kg after 96 h;^32^ for reproductive effects, a LOEC (lowest observed effect concentration) of 0.94 mg/kg after 28 days;^33^ and for sediment avoidance response, an AC50 (avoidance concentration 50%) of 0.19 mg/kg after 48 h.^32^ We aimed to gain a deeper understanding of how fluoxetine affects *L. variegatus* across different biological levels at environmentally relevant concentrations.

The following research questions were chosen for this study: 1) Does chronic fluoxetine exposure affect biochemical, physiological, and behavioral endpoints in *L. variegatus*? 2) Do these effects occur at environmentally relevant concentrations? 3) Are NCEs (molecular, physiological, and behavioral) more sensitive than conventional endpoints (mortality, growth, reproduction)? Moreover, 4) can a qualitative AOP connect molecular- and cellular-level effects to individual-level responses?

To answer these questions, two 28-day experiments were conducted at environmentally relevant fluoxetine concentrations, in accordance with OECD 225,^12^ with modifications, and the results were integrated into a qualitative AOP.

## 2 Materials and Methods

### 2.1 Culture Conditions of *Lumbriculus variegatus* and Preparation for Testing

At the start of the project, individuals of the species *Lumbriculus variegatus* were obtained from a commercial supplier (NatureHolic GmbH, Germany), as laboratories referenced in OECD Guideline 225 no longer maintained this species. The previous contaminant exposure history of the organisms is unknown; therefore, they cannot be considered demonstrably naïve. Prior to the experiments, organisms were acclimatized in the laboratory for several months. Cultures were maintained at CESAM, University of Aveiro, in 5 L glass tanks at 20 ± 2 °C, with a 16:8 light/dark cycle, under semistatic, aerated conditions, following OECD 225.^12^ Tanks contained a 2–3 cm layer of commercially available river sediment (<1 mm; previously washed with distilled water and burned at 500 °C to remove all organic matter) and American Society for Testing and Materials (ASTM) hard water^34^ (pH 7.6 ± 0.3). In the cultures, water was renewed twice a week, and organisms were fed powdered macerated fish food—TetraMin flakes (Tetra Werke, Melle, Germany; protein content ∼46%) (0.8 mg per cm^2^ of sediment surface in the culture vessel).

Since *L. variegatus* reproduces solely asexually via architomy in the laboratory, worms were synchronized 14 days before exposure,^12^ meaning individuals (4–5 cm, unfragmented) were cut in half to standardize the life stage. Tail segments were maintained in a separate aquarium and, once head regeneration was complete, fed TetraMin every 3 days and transferred to the exposure system 24 h after sediment spiking. All culture-handling and exposure experiments were conducted in accordance with established ecotoxicological guidelines to ensure minimal distress.

### 2.2 Chronic Exposure

Given the broad range of endpoints and high replication required for the behavioral tests, two chronic experiments were conducted (Figure 1). Chronic I assessed mortality, reproduction, oxidative stress/damage (CAT, GST, TG, LPO), neurotoxicity (ChE), energy metabolism (proteins, sugars, lipids, and ETS), and chemical avoidance. Chronic II assessed mortality, reproduction, growth, oxygen consumption, phototaxis, and feeding.

**Figure 1:**
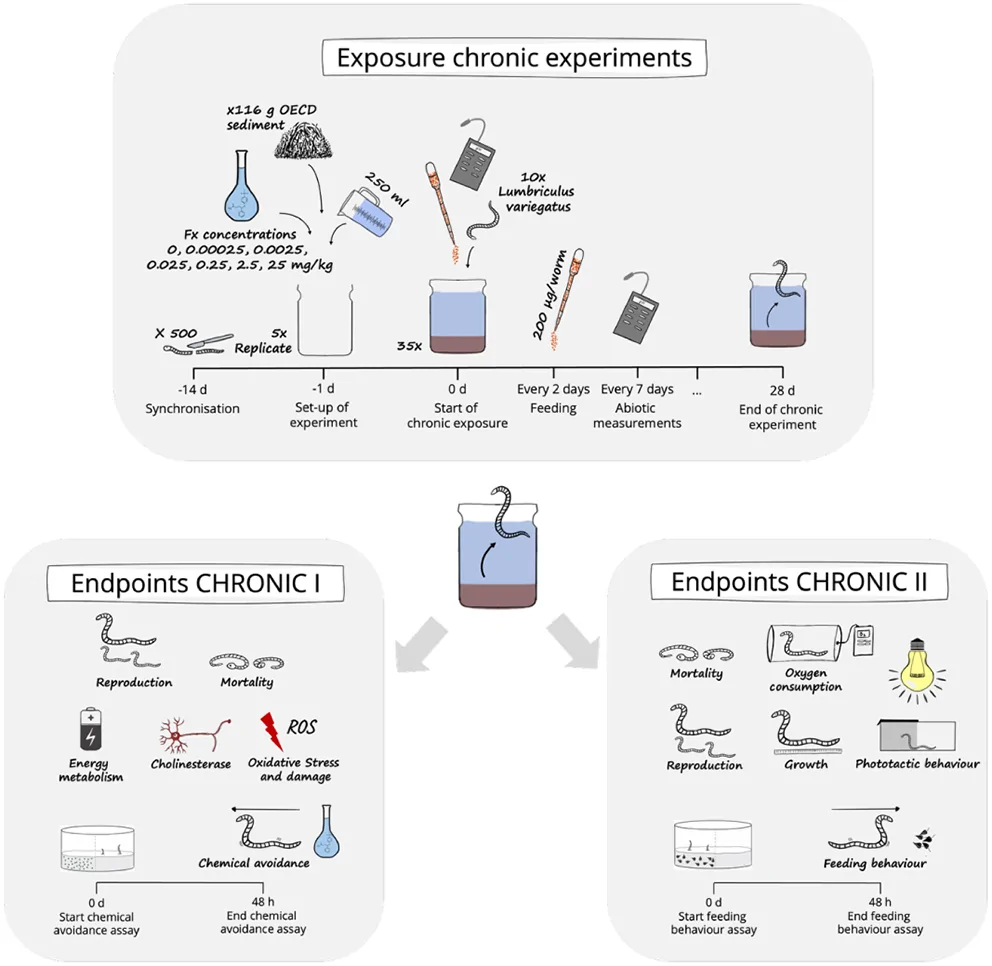
Experimental design of chronic experiments with *Lumbriculus variegatus* exposed to fluoxetine. Two 28-day exposures following OECD 225, followed by sampling for various endpoints and another 48 h exposure to assess chemical avoidance and feeding behavior (illustrated by Jacqueline Hilgendorf).

Following OECD 225, the sediment consisted of 75% sand (of which 50% was coarse sand (<1 mm) and 50% fine sand (<0.2 mm)), 20% kaolin, 4.5% peat (Siro Turfa, Leal & Soares S.A., pH 5.5–6.5), and 0.5% powdered alder leaves (food source). For each replicate, 80 g of dry sediment homogenized with 36 mL of ultrapure water (representing 45% of the sediment’s dry weight (DW)) was used.

Sediment was spiked in bulk for each treatment as recommended by OECD 225. Fluoxetine (CAS No. 56296-78-7; Sigma-Aldrich; purity ≥ 98%) was dissolved in ultrapure water to prepare a 300 mg/L stock solution. Working solutions were used to spike sediments by adding 6 mL per replicate (adding up to 52.5% of the sediment’s DW). Controls received 6 mL of ASTM water. Seven treatments (six fluoxetine concentrations plus a control) were tested with nominal concentrations of 0, 0.00025, 0.0025, 0.025, 0.25, 2.5, and 25 mg fluoxetine/kg DW sediment (Table 1 for measured concentrations). Five replicates were used for the fluoxetine exposure treatments and ten for the control, as extra organisms were needed specifically for the validation of the behavioral experiments. For each treatment, three additional replicates without worms were prepared for the chemical analysis. Fluoxetine concentrations in our study span environmentally relevant (0.00025–0.025 mg/kg in sediment DW)^28–30^ to high concentrations (up to 25 mg/kg) to evaluate dose–response relations and to maximize the chance of accurately detecting the effect thresholds for multiple target endpoints.

**Table 1:** Measured Concentration of Fluoxetine in the First (Chronic I) and Second (Chronic II) Experiments

**Nominal Concentrations** (mg/kg)	**Measured Sediment Concentrations** (mg/kg)			**Measured Pore Water Concentration** (mg/L)	**Measured Overlying Water Concentration** (mg/L)
**Chronic I+II**	**Chronic I**	**Chronic II**		**Chronic II**	**Chronic II**
	**End**	**Beginning**	**End**	**End**	**End**
0	<LOD^a^	<LOD^a^	<LOD^a^	<LOD^b^	<LOD^b^
0.00025	<LOD^a^	<LOD^a^	<LOD^a^	<LOD^b^	<LOD^b^
0.0025	<LOD^a^	<LOD^a^	<LOD^a^	<LOD^b^	<LOD^b^
0.025	0.018 ± 0.001	0.017 ± 0.001	0.018 ± 0.001	<LOD^b^	<LOD^b^
0.25	0.09 ± 0.01	0.06 ± 0.01	0.065 ± 0.002	<LOD^b^	<LOD^b^
2.5	1.03 ± 0.03	1.29 ± 0.04	1.3 ± 0.1	<LOD^b^	0.0455 ± 0.0007
25	11 ± 1	6.2 ± 0.2	12.4 ± 0.2	0.08 ± 0.02	0.15 ± 0.05

a LOD (Limit of detection) for sediment: 0.017 mg/kg.

b LOD for water: 0.014 mg/L.

After mixing, 122 g of spiked sediment was placed in 200 mL glass vials. 125 mL of ASTM water was added to each vial, and the vials were left for 24 h to equilibrate and allow sediment settling. Exposure was then initiated with aeration and the addition of ten synchronized worms per replicate. Worms transferred showed no recent fragmentation. Vials were maintained at 20 °C, 16:8 h light:dark, and 100–500 lx under gentle aeration for 28 days. In addition to the alder leaves in the sediment, the worms were fed TetraMin every 2 days (200 μg/worm; dissolved in ASTM water and added to the water surface with a pipette). Abiotic parameters (pH, conductivity, ammonia, and oxygen levels) were monitored weekly.

After 28 days, adult and offspring worms were retrieved, counted, and pooled for biochemical, behavioral, oxygen consumption, and growth assessments (Figure 1). Pooling was performed within replicates only, with organisms from a single replicate used for each assessment. Offspring were only used for follow-up behavioral tests if they had already regenerated key anatomical structures, namely the head and tail tip, ensuring the presence of sensory structures required to respond to environmental stimuli. Sediment samples were taken for chemical analysis at the end of Chronic I and at the beginning and end of Chronic II, then stored at −20 °C. Overlying water and pore water samples were taken at the end of Chronic II and also stored at −20 °C.

### 2.3 Biochemical Measurements

After exposure in Chronic I, six pooled samples per treatment (seven individuals each) were stored at −80 °C for biochemical analysis. Three replicates per treatment for oxidative stress (CAT, GST, TG), damage (LPO), and neurotoxicity (ChE), and three replicates for energy metabolism (lipids, sugars, proteins, ETS). All samples were measured in duplicate. The biochemical analysis for oxidative stress and damage, as well as neurotoxicity, follows the protocol described by Campos et al.^32^ For endpoints related to energy metabolism, the protocol follows that described by Coen et al.,^35^ later modified by Rodrigues et al.^36^ and Silva et al.^37^ All measured biochemical markers were combined with the Integrated Biomarker Response version 2 (IBRv2).^38–40^ For full methods and IBRv2 calculation, see Supporting Information SI A1 and A2.

### 2.4 Oxygen Consumption

Oxygen consumption was measured using simple static respirometry with worms held for 24 h in 50 mL gastight syringes (Hamilton, USA), following Pestana et al.^41^ For each treatment, three syringes with five worms from Chronic II were prepared as replicates. Oxygen consumption was expressed as μg of oxygen consumed/mg of organism/hour. For a detailed description, see Supporting Information SI A3.

### 2.5 Behavioral Endpoints

Behavior was assessed after chronic exposure in three assays designed to evaluate the antidepressant’s effects on chemical and phototactic sensory organs: A) chemical avoidance test (48 h; Figure 2A), B) feeding behavior test (48 h; Figure 2B), and C) phototactic response test (10 min; Figure 2C), described below. All behavioral assays were conducted outside the exposure medium to avoid the confounding effects of fluoxetine on behavior (e.g., mixture toxicity).^32^

**Figure 2:**
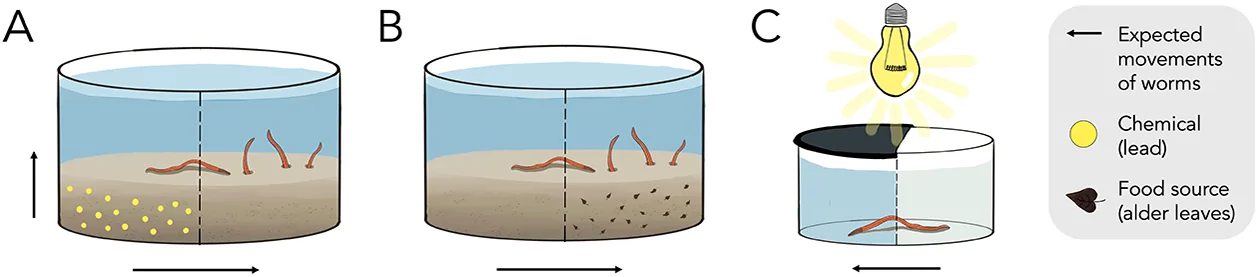
Schematic overview of *Lumbriculus variegatus* behavioral assays after 28 days of fluoxetine exposure. A) 48 h chemical avoidance of sediment contaminated with lead: sediment on one side of the test container is spiked with lead, the other side is filled with uncontaminated sediment. B) 48 h feeding behavior: sediment on one side of the test container is mixed with a food source (alder leaves), the other side is with plain sediment. C) 10 min phototactic behavior: the test container is exposed to a light source and covered with a lid, so one side is opaque (a proxy for shelter) while the other is exposed to light. Arrows indicate the direction of the worms’ expected movement (illustrated by Jacqueline Hilgendorf).

#### 2.5.1 Chemical Avoidance of Contaminated Sediment with Lead

To assess whether fluoxetine affected chemosensory function, worms previously exposed to fluoxetine in Chronic I were tested in a two-compartment chemical avoidance assay described by Hilgendorf et al.,^32^ using lead as a model contaminant (Figure 2A). Circular plastic containers (40 × 95 mm) were divided into equal compartments, each containing 55 g of either Pb-contaminated or clean sediment, with a plastic split separating the two compartments. Lead­(II) acetate trihydrate (CAS No. 6080-56-4) was dissolved in ultrapure water to create the stock solution for the contaminated sediment. The stock solution was diluted to 632 mg/kg (the previously determined AC_50_).^32^ Validation of the two-compartment assay required confirming homogeneous worm distribution in controls, ensuring movements reflected true avoidance rather than setup bias. Therefore, two controls were included: (1) uncontaminated sediment on both sides to validate the design (Ct–Ct), and (2) lead in only one compartment to verify stimulus detection.

After addition of the sediment, the plastic split was removed, and containers were gently filled with water to prevent mixing. Setups were equilibrated for 24 h to allow chemical distribution and sediment settling. Following this equilibration period, exposure was initiated by aerating and adding worms to each replicate. Worms from the chronic exposure treatments with fluoxetine and their respective negative control were transferred into the test containers (five replicates per treatment/ten worms per replicate). Only intact, nonfragmented worms were used, and they were placed in the middle to allow equal access to both compartments.

Test containers were incubated for 48 h at 20 °C, 16:8 h light:dark, 100–500 lx, under gentle aeration. After exposure, the plastic split was reinserted, and the worms were carefully removed and counted from each side to calculate avoidance behavior using the following equation:

1 Avoidance⁡(%)=(C−T)/N×100

C represents the number of worms in control sediment (including those on the surface or in the water column), T is the number in contaminated sediment, and N is the total number per replicate. Positive values indicated avoidance of contaminated sediment, negative values a preference, and zero a neutral distribution.^42^

#### 2.5.2 Feeding Behavior

Feeding behavior was assessed postexposure following Chronic II using a two-compartment assay (Figure 2B) following the chemical-avoidance protocol (Section 2.5.1). One side contained OECD sediment with 0.5% powdered alder leaves (food source), and the other contained plain sediment. Similar to the setup for the chemical avoidance behavior, two controls were used: (1) food on both sides to validate the design (Ct–Ct) and (2) food on one side only to confirm stimulus detection. In both cases, worms from the control group of Chronic II were used.

#### 2.5.3 Phototactic Behavior

Phototactic behavior was evaluated postexposure following Chronic II (Figure 2C). Fifty intact worms per treatment were individually placed in plastic containers (40 × 95 mm) with ASTM medium and exposed to light. Light was provided by a cold white LED lamp (806 lm, 6400 K) positioned 20 cm above the containers, yielding ∼1000 lx. Worms were placed on the illuminated side, and each container was then covered by a two-section lid: one side opaque (black tape) and the other transparent. Worm’s distribution was recorded at 2, 5, and 10 min by counting the number of individuals remaining in the light-exposed area. Avoidance was calculated as in Section 2.5.1 (eq 1).

Only one control condition (opaque on one side) was used. A control with the same condition on both sides was not feasible, as covering both sides made it impossible to determine the worm position without moving the container, biasing the results. Conversely, performing the assay without lids also introduced bias, as worms can become agitated and display excessive movement under light exposure.

### 2.6 Conventional Endpoints: Mortality, Reproduction, and Growth

Mortality and reproduction were measured postexposure after Chronic I and II, following OECD 225.^12^ We distinguished the worms by 1) adults: large, intact individuals with dark red anterior regions (fully developed adults) or without a lighter regenerated posterior (recently reproduced); 2) offspring (fully developed): small, complete individuals with overall lighter-colored body regions; and 3) offspring (recently fragmented): incomplete posterior fragments without head or tail. Furthermore, for the test to be considered valid, control reproduction must increase by a factor of 1.8, meaning that the initial count of 10 individuals should reach at least 18 by the end of the experiment.

Growth was determined after Chronic II as the DW of organisms in milligrams. Five worms (adults and offspring) were randomly pooled for five replicates per treatment, euthanized using ethanol, frozen at −80 °C, lyophilized, and weighed using a microscale.

### 2.7 Chemical Analysis

The chemical analysis followed the protocol described in detail in Hilgendorf et al.,^32^ including full analytical details and validation parameters. Briefly, fluoxetine in sediment was quantified by validated extraction and High-Performance Liquid Chromatography with Fluorescence Detection (HPLC-FLD). For this, 5 g of the sample was extracted with methanol containing 2% NH_4_OH, and the supernatants were analyzed on a Shimadzu LC-20AT Prominence HPLC system with fluorescence detection. Separation used a C18-PFP column and a mobile phase of acetonitrile:formic acid (0.1%) (45:55, v/v) at a flow rate of 0.7 mL/min. Two calibration curves covered low (0.1–5 mg/kg; r^2^ = 0.9981) and high (2.5–250 mg/kg; r^2^ = 0.9997) concentration ranges. The limit of detection (LOD) was 0.017 mg/kg.

### 2.8 Statistical Analysis

Statistical analyses were performed in GraphPad 10.2.0 with significance at *p* < 0.05. Data normality and homoscedasticity were tested using Shapiro–Wilk and Bartlett’s tests, respectively. The lipid and protein data did not meet the assumptions of normality and homoscedasticity and were analyzed with the Kruskal–Wallis test. All other endpoints met assumptions and were analyzed by one-way ANOVA. Significant differences were identified with Dunnett’s post hoc test (control vs treatments) or Tukey’s test (between treatments). We used the lowest observed effect concentration (LOEC) to report significant effects in the qualitative AOP (Figure 7). LOECs and NOECs (no-observed effect concentrations) are reported in Table S3 (Supporting Information).

As noted in Sections 2.5.1 and 2.5.2, control worms were tested for homogeneous distribution to validate the two-compartment design and exclude setup bias. Distributions were analyzed with Fisher’s Exact Test (*n* = 50). The test was also used to determine whether worms exposed to fluoxetine treatments significantly avoided/preferred one site in the sediment avoidance, feeding, and phototactic behavior test. This was followed by ANOVA to compare responses among treatments.

## 3 Results

Tests were conducted according to OECD 225 ^12^ with modifications and met validity criteria: pH 6–9, dissolved oxygen >30% saturation (Tables S1 and S2), no mortality in controls, and normal burrowing behavior. No mortality occurred in any replicate of the chronic or behavioral assays, confirming that the test conditions were valid and reliable. Regarding reproduction, control organisms met the OECD validation criterion (reproduction rate of 1.8)^12^ in both chronic experiments, with an average reproduction of 3.6–3.7.

### 3.1 Chemical Analysis

The results of the chemical analysis for measured fluoxetine concentrations in sediment from both chronic experiments, as well as the pore water and overlying water concentrations from Chronic II, are presented in Table 1. Overall, the sediment matrix was successfully contaminated, with fluoxetine reliably quantified from 0.025 mg/kg upward, confirming an increasing concentration of fluoxetine with increasing exposure dose.

At the lowest nominal concentrations (0.00025 and 0.0025 mg/kg), fluoxetine was not detected, as the concentrations were below the method’s limit of detection (LOD = 0.017 mg/kg). At the nominal concentration of 0.025 mg/kg, measured values were 0.018 ± 0.001 mg/kg in both experiments, corresponding to approximately 72% of fluoxetine adsorbed. For the 0.25 mg/kg treatments, fluoxetine adsorption onto the sediment ranged from 24% to 36%, with measured concentrations between 0.06 ± 0.01 and 0.09 ± 0.01 mg/kg. At 2.5 mg/kg, fluoxetine levels in sediment ranged from 1.03 ± 0.03 to 1.3 ± 0.1 mg/kg, corresponding to sediment uptake of 41% to 52%. The highest nominal concentration (25 mg/kg) resulted in measured concentrations of 6.2 ± 0.2 mg/kg at the beginning and 12.4 ± 0.2 mg/kg at the end of Chronic II, representing a 50% sediment uptake. Since the two lowest concentrations could not be analytically detected, we present the Results and Discussion as nominal concentrations.

Measured concentrations of fluoxetine in the overlying water were below the limit of detection (LOD; 0.014 mg/L) in all treatments except the two highest, which accounted for 1–3% of the total chemical mass. Pore water concentrations were only quantifiable in the highest treatment and accounted for less than 1%.

### 3.2 Biochemical Endpoints

#### 3.2.1 Integrated Biomarker Response

Biochemical responses were integrated using the biomarker deviation index (A) and visualized as star plots (Figure 3A: energy metabolism; Figure 3B: oxidative stress and damage and neurotoxicity).^40^ Star plots show deviations from controls (dashed lines) across fluoxetine concentrations. The IBRv2, which represents the summed absolute values of A calculated for each biomarker and each fluoxetine treatment, also represented as the area of the star plots, is shown in Figure S3. No significant variation in energy metabolism was observed except for ETS, whereas oxidative stress and damage showed dose-dependent increases from 0.025 mg/kg upward; the neurotoxicity biomarker (ChE) showed no significant change (Figure S3). Individual biomarker data are provided in Figure S1.

**Figure 3:**
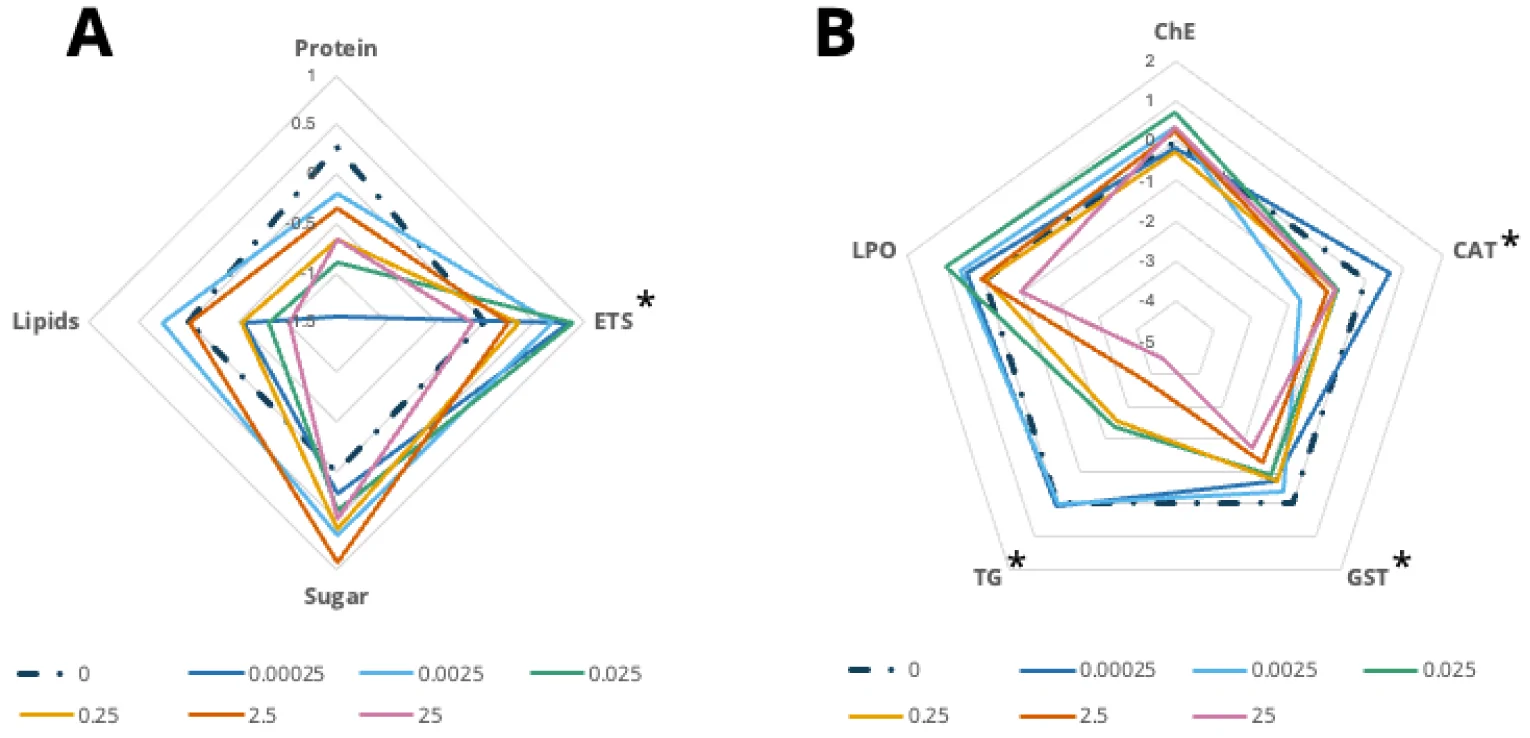
Biochemical responses in *Lumbriculus variegatus* after 28 days of chronic exposure to fluoxetine, represented through the Integrated Biomarker Response (IBRv2). A) Energy metabolism (proteins, sugars, lipids, ETS). B) Oxidative stress (CAT, GST, TG) and damage (LPO), and neurotoxicity (ChE). Data are normalized, and star plots are used to visualize deviations from the control (CTR = 0, dashed line). Asterisks (*): significant differences from the control group (one-way ANOVA, Dunnett’s test, *p* < 0.05).

#### 3.2.2 Energy Metabolism

Among metabolic biomarkers, only ETS activity showed a significant effect after fluoxetine exposure (one-way ANOVA, F_6,35_ = 6.361, *p* = 0.0001). Only organisms at the highest concentration (25 mg/kg) were significantly affected compared with the control (Dunnett’s test, *p* = 0.03). However, ETS activity increased at the first three fluoxetine concentrations and then decreased at the last three (Figure 3A and Figure S1A). When comparing the exposure treatments, a significant difference was observed between the first three concentrations and 25 mg/kg (Tukey’s test, *p* < 0.05), with the greatest difference between 0.025 and 25 mg/kg (Tukey’s test, *p* = 0.0003).

Fluoxetine exposure did not significantly affect the worms’ protein (Kruskal–Wallis test, *p* > 0.05), lipid (Kruskal–Wallis test, *p* > 0.05), or sugar contents (one-way ANOVA, F_6,35_ = 0.8908, *p* > 0.05) (Figure 3A and Figure S1B–D).

#### 3.2.3 Neurotoxicity

The ChE activity of *L. variegatus* from the negative controls averaged 294 nmol/min/mg protein (±31), with fluoxetine exposure not inducing any significant effects (Figure 3B and Figure S1E; one-way ANOVA, F_6,35_ = 1.194, *p* > 0.05).

#### 3.2.4 Oxidative Stress and Damage

Measured oxidative stress and damage markers were significantly affected by fluoxetine exposure, although responses varied by biomarker (Figure 3B and Figure S1). CAT activity was significantly impacted (one-way ANOVA, F_6,35_ = 6.138, *p* = 0.0002). It was the most sensitive marker, showing a significant increase, but only at 0.00025 mg/kg (Dunnett’s test, *p* = 0.01) (Figure S1G). TG was also significantly affected (one-way ANOVA, F_6,35_ = 28.03, *p* < 0.0001) and identified as the second most sensitive marker, showing a significant decrease at all tested concentrations, except for the two lowest (Dunnett’s test, *p* < 0.0001) (Figure S1I). GST was also significantly affected (one-way ANOVA, F_6,35_ = 2.545, *p* = 0.0376), with a significant decrease at the two highest concentrations (Dunnett’s test, *p* < 0.05) (Figure S1H). LPO showed a general difference among treatments (one-way ANOVA, F_6,35_ = 3.203, *p* = 0.01), but no significant difference was observed for any treatment compared to the control (Dunnett’s test, *p* > 0.05). There was, however, a significant difference between 0.025 and 25 mg/kg (Tukey’s test, *p* = 0.006) as LPO concentrations showed a slight increase at the first three concentrations, followed by a decrease at the last three concentrations (Figure 3B and Figure S1F).

### 3.3 Oxygen Consumption

Oxygen consumption averaged 28 μg O2/mg organism/h (±3.6) in controls, and no significant differences among treatments were observed (one-way ANOVA, F_6,13_ = 0.4011, *p* > 0.05; Supporting Information, Figure S2).

### 3.4 Behavioral Endpoints

#### 3.4.1 Validation of Behavioral Assays

Organisms exhibited no directional preference, distributing homogeneously across both control compartments (Ct–Ct), confirming the validity of the experimental design for chemical avoidance and feeding behavior (Fisher’s Exact Test, *p* > 0.05, *n* = 50).

#### 3.4.2 Chemical Avoidance of Contaminated Sediment with Lead

The fluoxetine control group showed a significant avoidance of lead-contaminated sediment (Fisher’s Exact Test, *p* < 0.05; *n* = 50), confirming the assay’s sensitivity. Previously experienced fluoxetine exposure significantly altered the worms’ avoidance behavior (one-way ANOVA, F_7,30_ = 2.645, *p* = 0.03). However, only worms previously exposed to 0.025 mg/kg showed a significant difference from the control (Figure 4A; Dunnett’s test, *p* = 0.02), displaying a preference for the lead-contaminated sediment.

**Figure 4:**
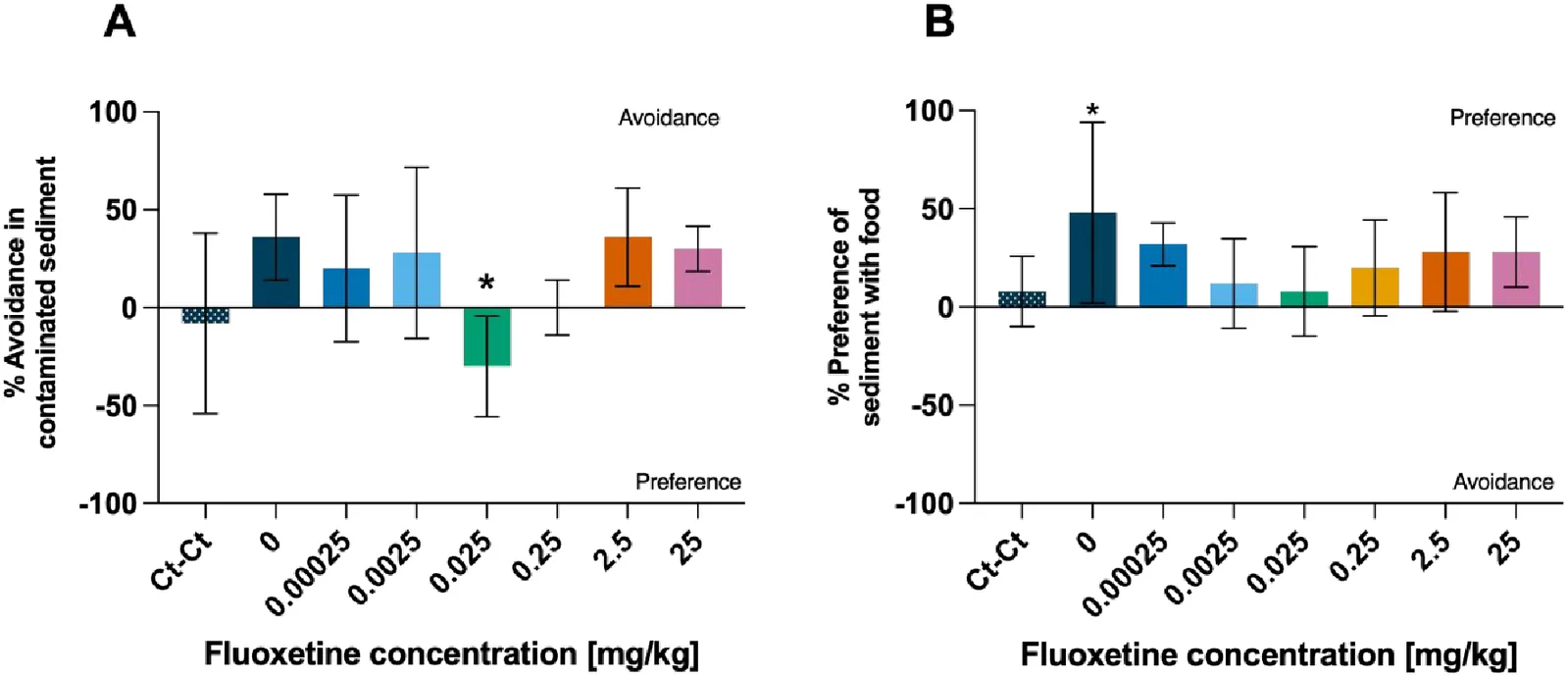
Behavioral responses of *Lumbriculus variegatus* pre-exposed to fluoxetine for 28 days, expressed as mean ± SD. A) Lead avoidance assay in a two-compartment system with sediment for 48 h (632 mg Pb/kg). B) Feeding behavior assay in a two-compartment system (food vs no-food) with sediment for 48 h. Positive values indicate preference for sediment without lead/with food, while negative values indicate avoidance. Asterisks in A (*): significant differences from control organisms of the fluoxetine exposure (one-way ANOVA followed by Dunnett’s test, *p* < 0.05). Asterisks in B (*): Ct–Ct: validation treatment with controls on both sides.

#### 3.4.3 Feeding Behavior

Control worms from the fluoxetine experiment displayed a significant preference for the food-containing compartment (Fisher’s Exact Test, *p* = 0.01; *n* = 50), suggesting that unexposed individuals could detect and actively move towards food. Worms previously exposed to fluoxetine showed only a weak tendency to prefer the food compartment (Figure 4B), but none of the treatment groups exhibited a statistically significant response (Fisher’s Exact Test, *p* > 0.05). The overall ANOVA results showed no significant differences among treatments (one-way ANOVA, F_(7, 52)_ = 1.355, *p* > 0.05).

#### 3.4.4 Phototactic Behavior

The results of the Fisher Exact Test indicated that control worms significantly avoided the illuminated area after 2 and 5 min, validating the behavioral assay design (Fisher’s Exact Test, *p* < 0.05, *n* = 50). This avoidance response was no longer observed after 10 min, as the worms appeared to acclimate to the environment and exhibited more exploratory behavior (Fisher’s Exact Test, *p* > 0.05, *n* = 50).

Worms previously exposed to fluoxetine exhibited a markedly different pattern with no significant light avoidance at any concentration or time point (Fisher’s Exact Test, *p* > 0.05, *n* = 50). Worms exposed to 0.0025 mg/kg fluoxetine displayed a significant preference for the illuminated area after 2 min (Fisher’s Exact Test, *p* = 0.03, *n* = 50), suggesting a disruption or reversal of the typical avoidance response.

ANOVA results further revealed that fluoxetine exposure significantly altered worm behavior compared to controls after 2 min (one-way ANOVA, F_6,28_ = 6.701, *p* = 0.0002) and 5 min (one-way ANOVA, F_6,28_ = 2.650, *p* = 0.04). After 2 min, all fluoxetine treatments showed significantly reduced avoidance responses compared with the control treatment (Dunnett’s test, *p* = 0.001; Figure 5A). After 5 min, worms previously exposed to 0.0025, 0.025, and 0.25 mg/kg fluoxetine still showed significantly altered behavior compared with controls (Dunnett’s test, *p* < 0.05; Figure 5B). However, the worm’s behavior in the other treatments became less distinct as more individuals avoided the illuminated area. By 10 min, no significant differences were observed across any treatments, as control worms also ceased to avoid the light (one-way ANOVA, F_6,28_ = 0.5614, *p* > 0.05; Figure 5C).

**Figure 5:**
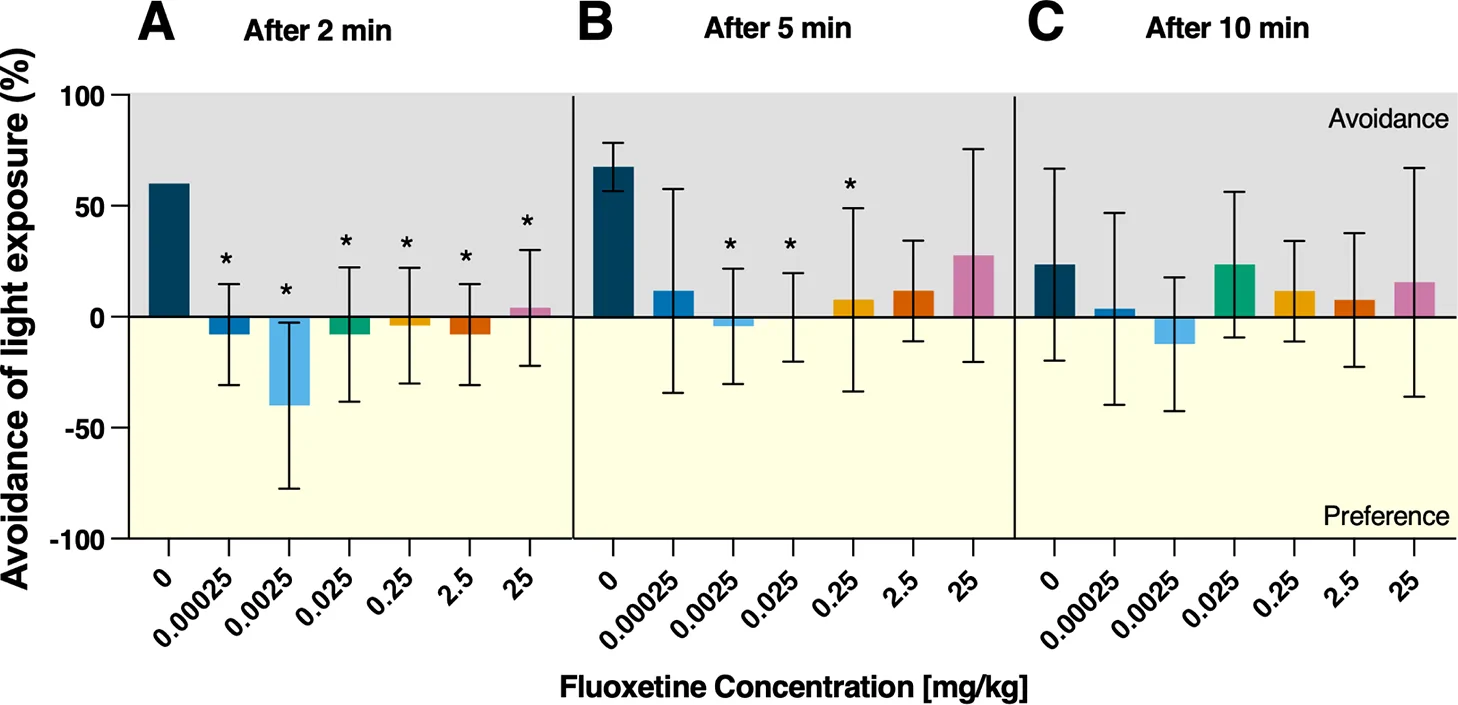
Phototactic responses of *Lumbriculus variegatus* measured after 28 days of chronic exposure to fluoxetine after 2 (A), 5 (B), and 10 min (C) of light exposure. Avoidance: organisms moved out of the light into the shadow area (grey background). Preference: organisms stayed in the light area (yellow background). Data are presented as mean ± SD. Asterisks (*): significant differences from the respective control (one-way ANOVA, Dunnett’s test, *p* < 0.05).

### 3.5 Conventional Endpoints: Mortality, Reproduction, and Growth

No mortality was observed in any of the controls or fluoxetine-treated groups. However, fluoxetine exposure resulted in a statistically significant reduction in organism growth compared to the control in Chronic II (one-way ANOVA, F_6,14_ = 4.722, *p* = 0.008). Significant effects were observed at concentrations of 0.0025, 0.25, 2.5, and 25 mg/kg (Dunnett’s test, *p* < 0.05) but not at the intermediate concentration of 0.025 mg/kg (Dunnett’s test, *p* > 0.05) (Figure 6A). Mean organism DW declined by 42%, from 2.4 ± 0.25 mg (control) to 1.4 ± 0.46 mg (25 mg/kg) per worm.

**Figure 6:**
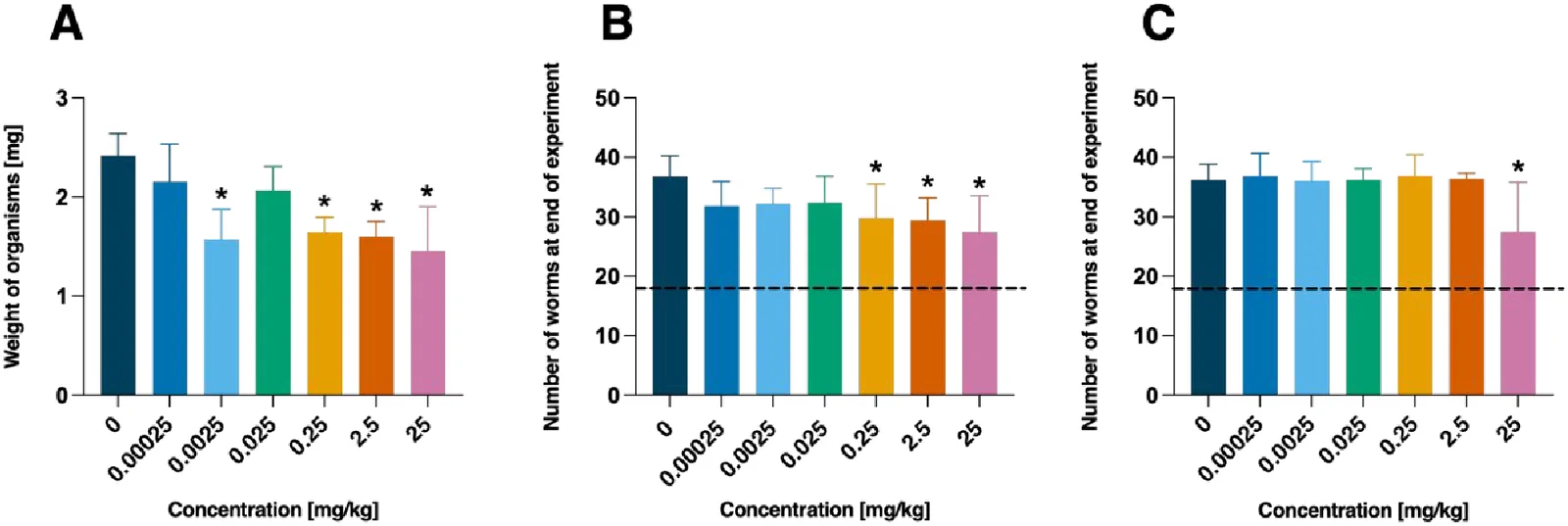
A) *Lumbriculus variegatus* growth expressed as the mean individual DW (mg) (Chronic II). B) and C) Reproduction of *L. variegatus* in Chronic I and Chronic II as the number of worms at the end of the experiment (mean ± SD). Horizontal line: validation criterion (reproduction rate of 1.8). Asterisks (*): statistically significant differences compared to the control group (one-way ANOVA, Dunnett’s test, *p* < 0.05). *n* = 10 for control; *n* = 5 for exposure treatments.

Regarding reproduction, control organisms met the OECD validation criterion (reproduction rate of 1.8)^12^ in both chronic experiments, with average reproduction rates of 3.7 in Chronic I and 3.6 in Chronic II (Figure 6B and C). Exposure to fluoxetine significantly impaired reproduction in Chronic I (one-way ANOVA, F_6,28_ = 2.862; *p* = 0.03) and Chronic II (one-way ANOVA, F_6,33_ = 3.797; *p* = 0.005), although significant effect concentrations differed between experiments. In Chronic I, reproduction was inhibited at the three highest concentrations (0.25, 2.5, and 25 mg/kg) compared to the control (Dunnett’s test, *p* < 0.05, Figure 6B). The final average worm count was reduced by 30%, from 36.7 ± 6 worms (control) to 27.4 ± 10 worms (25 mg/kg). In Chronic II, reproduction was only reduced in the highest concentration (25 mg/kg) (Dunnett’s test, *p* = 0.002, Figure 6C). However, similar to Chronic I, reproduction was reduced by 25%, from 36.2 ± 4 worms (control) to 27.4 ± 12 worms (25 mg/kg). In Figure S4, reproduction data are distinguished between adults and offspring, showing that many offspring were produced shortly before the end of the experiment (no body part regeneration has been observed yet).

### 3.6 Integration of All Endpoints with Adverse Outcome Pathway

As defined for endocrine disruptors, assessing a hazardous chemical requires measuring molecular and cellular events, evaluating adverse effects, and establishing a “biologically plausible link” between them.^43^ We adapted this approach in our study to measure the effects of the neuroendocrine chemical fluoxetine. AOPs are recommended to link the molecular initiating event (MIE) with measured key events (KEs) at the molecular/cellular/organ levels to the adverse outcomes (AOs) at the individual/population levels. We did not measure serotonin levels and, therefore, cannot confirm a causal link between the MIE—serotonin transporter inhibition—and the observed effects at higher biological levels. However, fluoxetine can modulate serotonin activity in aquatic invertebrates,^9^ and *L. variegatus* possesses serotonin receptors and endogenous serotonin.^21,23^ Given that we observed effects across all biological levels following fluoxetine exposure, we propose serotonin transporter inhibition as the potential MIE. We developed a qualitative AOP for the effects of fluoxetine in *L. variegatus*, based on our integrated findings (Figure 7), presenting LOECs when significant effects were observed. For a more detailed description of the AOP construction, see Supporting Information SI A4.

**Figure 7:**
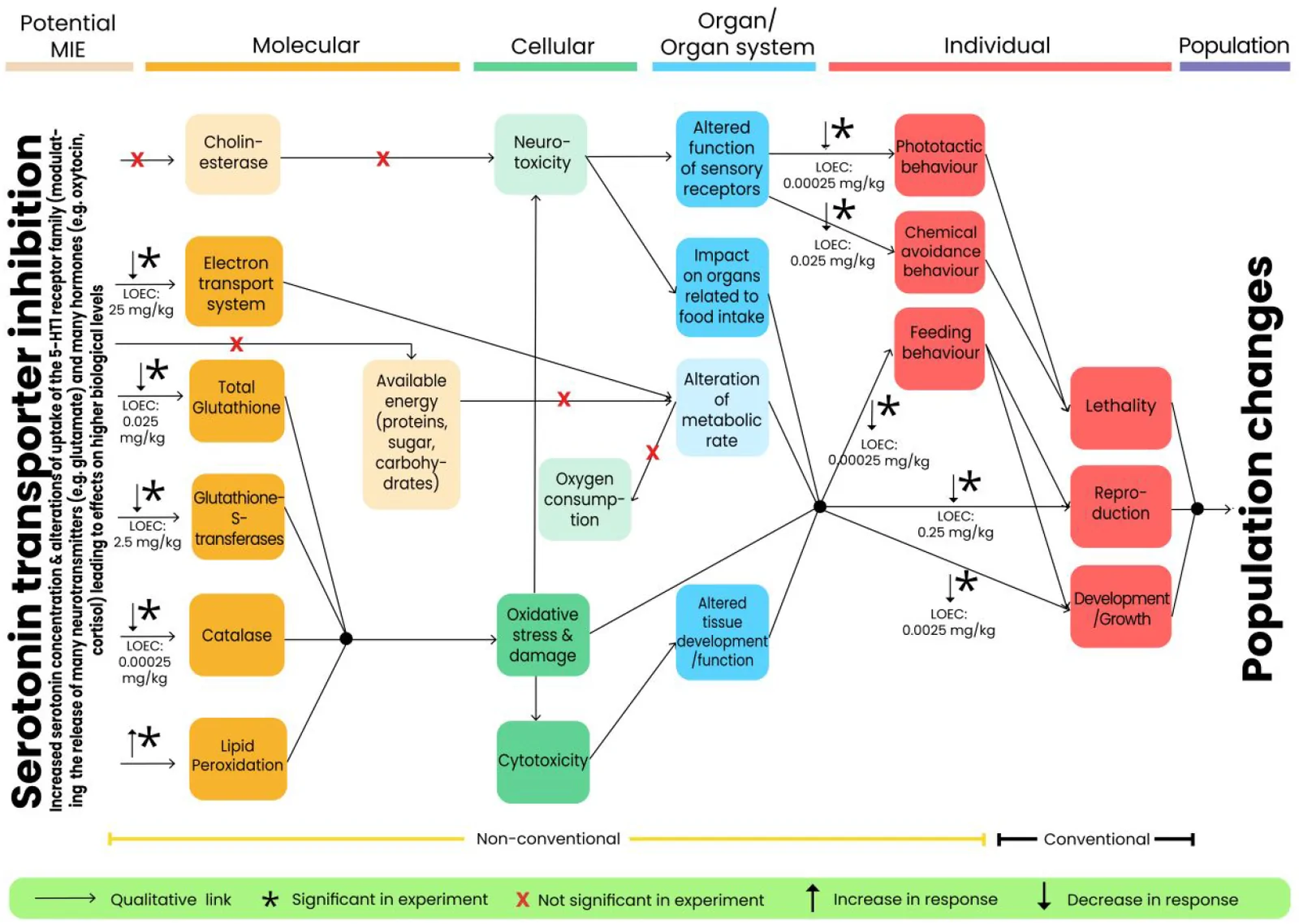
Qualitative Adverse Outcome Pathway (AOP) for fluoxetine in *Lumbriculus variegatus*. The diagram illustrates the cascade of biological effects starting from the molecular initiating event (MIE)—inhibition of the serotonin transporter—through key events (KEs) at the molecular, cellular, and organ/system levels, ultimately leading to adverse outcomes (AOs) at individual/population levels. Solid lines: putative mechanistic links. Asterisks (*): endpoints with significant effects presented as LOECs (Lowest Observed Effect Concentrations). Arrows (↑ ↓): endpoint increase or inhibition. Red crosses (x) and lighter colors: endpoints measured but not significantly affected.

## 4 Discussion

Our study links mechanistic insights of sediment-borne fluoxetine exposure in *L. variegatus* to population-relevant effects by integrating biochemical, physiological, and behavioral endpoints within a qualitative AOP framework.

Measured fluoxetine levels ranged from 24% to 72% of the nominal values, reflecting the sediments’ adsorption capacity, which varies with the experimental conditions. The differences between the beginning and end reflected the slow uptake by the sediment and higher adsorption for longer contact times. This effect was especially pronounced at higher concentrations because the sediment had not reached adsorption equilibrium at the start of the experiment. At the end of the experiment, sediment adsorption reached values as high as ∼50% of the nominal concentration, consistently verified for Chronic I and Chronic II.

Despite the relatively short half-life of fluoxetine in the water column (1–4 days^44^), fluoxetine concentrations of up to 0.15 mg/L were detected in the overlying water at the end of the 28-day exposure. The bioturbation activity of *L. variegatus* may have caused the remobilization of fluoxetine from the sediment into the overlying water, as reported for other chemicals in previous studies.^45^ Consequently, exposure via the overlying water, pore water, and fluoxetine adsorbed to TetraMin may have contributed to the observed effects in *L. variegatus*. However, studies on *L. variegatus* and other sediment-dwelling worms (e.g., *Capitella teleta*) have shown that ingestion of contaminated sediment and dietary uptake represent major routes of chemical exposure.^46–48^ Therefore, we consider sediment-bound fluoxetine to be the primary driver of the observed effects in *L. variegatus*.

In the qualitative AOP, the proposed MIE is the inhibition of the serotonin transporters, leading to an increase in cellular serotonin. We did not measure serotonin levels in *L. variegatus* in this study, as no standardized method for our model organism was available. However, in a subsequent study, we adapted an ELISA (enzyme-linked immunosorbent assay) protocol for aquatic invertebrates and quantified serotonin in both control organisms and those exposed to fluoxetine via short-term water exposure (0, 10, and 100 μg/L).^22^ Although serotonin was successfully quantified, no significant effects of fluoxetine exposure were observed, despite a slight increasing trend with concentration. This apparent lack of response may reflect methodological constraints, such as the exposure medium, duration, or statistical power. Therefore, while serotonergic pathways remain a plausible target of fluoxetine, the present study does not provide direct evidence of serotonin modulation, and the mechanistic interpretation should be considered indirect. Furthermore, while fluoxetine primarily targets the serotonin system, additional molecular targets in aquatic invertebrates have been proposed, including dopamine transporters, 5-HT_2_ receptors, sigma receptors, muscarinic cholinergic receptors, and cytochrome P450 enzymes.^49^ Alterations in these molecular targets may also contribute to the observed effects in *L. variegatus*; however, further research is required to verify this.

The induction of the MIE can lead to molecular/cellular-level alterations, such as oxidative stress. Fluoxetine can traverse mitochondrial membranes and accumulate within the mitochondrial matrix, triggering mitochondrial calcium overload and increased reactive oxygen species (ROS),^50^ which in turn activate antioxidant defense mechanisms through hormesis, as observed, for instance, in the marine rotifer *Brachionus koreanus* ^51^ or the clam *Corbicula fluminea*.^52^ In our study, fluoxetine also induced significant oxidative stress, with CAT and TG being the most sensitive endpoints and GST the least. This reflects their physiological roles, as CAT and TG mediate the primary defense against ROS, while GST acts in the second phase of xenobiotic detoxification.^53^ ROS levels can return to baseline after acute oxidative stress if the organism’s antioxidant defenses remain robust. However, if the antioxidant system fails to neutralize excess ROS, the organism may enter a state of chronic oxidative stress, followed by oxidative damage, such as to cell membrane lipids, as measured by the LPO assay.^53^ We observed a slight increase in LPO, which diminished at higher concentrations. At the highest concentration, where we have also found the highest activity of xenobiotic-excreting enzymes, the organisms appear to cope better with ROS than at lower concentrations. Prolonged ROS imbalance can cause cytotoxicity and disrupt tissue development and function,^54,55^ leading to effects at higher biological levels (Figure 7).

For *L. variegatus*, a functional oxidative stress response is crucial, as ROS play a significant role in the worms’ regenerative ability. Although ROS are commonly considered toxic byproducts of cellular metabolism, at regulated concentrations, they also play important roles in processes such as wound healing and the regulation of cell proliferation, an important step in the regeneration process of *L. variegatus*.^56^ Chemical inhibition of ROS can delay regeneration^56^ and directly affect higher biological levels by reducing reproductive success. After 28 days, we still observed a shift in the antioxidant system, suggesting that the oxidative stress contributed to adverse reproductive effects in *L. variegatus*.

Another effect at the molecular/cellular level can be the depletion of energy reserves due to higher energy demands.^57^ Significant alterations in energy metabolism were observed in fish after fluoxetine exposure to similar experimental concentrations and durations^45^, and in the marine worm *Hediste diversicolor* after short-term exposure.^8^ Although we observed oxidative stress, energy metabolism remained largely unchanged, except for ETS activity. The lack of measurable changes might reflect the highly nutritious TetraMin diet, which may have helped the worms maintain their energy reserves^58^ and enhance their tolerance to chemical stress^59^ by neutralizing ROS and diminishing the effects on LPO. Another explanation, based on the Dynamic Energy Budget (DEB) theory, is that the chemical stressor increased maintenance costs required to preserve cellular energy homeostasis, thereby keeping energy reserves stable while reducing the energy available for growth and reproduction at higher concentrations.^60^

In our AOP, we hypothesized that increased cellular energy demands would be reflected in elevated ETS activity and, consequently, increased whole-organism oxygen consumption, as described by Hird et al.,^9^ who reported a LOEC of 10 μg/L after 72 h of waterborne fluoxetine exposure in *H. diversicolor*. Despite an increase in ETS activity at an intermediate concentration, oxygen consumption remained unaffected. One possible explanation is that we measured oxygen consumption postexposure, whereas Hird et al.^9^ did so during exposure. Another explanation might be the exposure medium, as they used water exposure to measure oxygen consumption. Over the longer exposure period, worms may have also adapted to the experimental conditions, or species-specific traits may have influenced the response. As a sediment-dweller adapted to environments with fluctuating oxygen levels, such as ponds that occasionally dry out,^61^ *L. variegatus* possesses metabolic adaptations, including high glycogen storage and the ability to sustain energy production under anoxic conditions.^62^ These traits allow the organism to maintain metabolic function without substantial changes in oxygen consumption, even under stress conditions.

Another measured endpoint at the molecular/cellular level was cholinesterase activity. While cholinesterase is a widely used biomarker of neurotoxicity, it does not directly reflect the primary mode of action of fluoxetine, which involves inhibiting the serotonin reuptake. Instead, it was included to capture potential broader neurophysiological disturbances. Interactions between serotonergic and cholinergic systems have been reported, and serotonin has been shown to modulate specific cholinesterase pools,^63^ although the extent and relevance of this interaction may vary across species. In *L. variegatus*, the interplay between these systems may be particularly relevant, as it has been hypothesized that the architomy reflex is initiated through cholinergic activation of serotonergic neurons.^23^ Despite this, there was no significant effect of fluoxetine exposure on ChE activity. Acetylcholinesterase (AChE) is the primary ChE type measured as a biomarker of neurotoxicity,^64^ and previous studies have reported significant alterations following short-term exposure to fluoxetine in the fish *Danio rerio* ^65^ and *Pomatoschistus microps*.^66^ However, AChE activity can fluctuate over time,^67^ and, as may have occurred in our study, effects could diminish with prolonged exposure. Moreover, the predominant ChE type varies among species, potentially leading to differences in sensitivities to contaminants.^68^ As *L. variegatus* possesses only atypical ChEs,^69^ these enzymes may be less susceptible to fluoxetine.

Other molecular changes can also induce neurotoxicity, such as oxidative stress,^64^ a highly sensitive marker for fluoxetine effects in our study. Additionally, serotonin, as a key neurotransmitter, plays a crucial role in neural function. Its receptors are involved in many physiological and behavioral systems, and their inhibition can lead to neurotoxic effects.^70^ This shows the need to measure multiple molecular and cellular endpoints to get an overall understanding of chemical effects. Therefore, we propose alternative pathways for neurotoxicity in our AOP, including oxidative stress and serotonin modulation.

These neurotoxic-related changes can affect organisms at the individual level, for instance, through behavioral changes. We observed fluoxetine-related effects across all three behavioral assays, significant alterations in phototaxis and chemical avoidance, and a loss of the food preference shown by controls, with important population-level implications, including altered sediment avoidance. Chemical avoidance tests assess an organism’s ability to detect and respond to contaminant exposure via chemoreceptor organs, thereby actively avoiding the contaminant. This is ecologically relevant, as impaired avoidance behavior reduces an organism’s capacity to escape chemical stressors, ultimately increasing the risk of population decline in the field.^71^ Our results indicate that prior exposure to fluoxetine affected the chemosensory organs, significantly reducing the worm’s ability to avoid lead-contaminated sediment. This is consistent with our prior study measuring the sediment-avoidance response of *L. variegatus* to fluoxetine and lead.^32^

All fluoxetine exposure treatments impaired feeding behavior related to food perception. In the control, *L. variegatus* actively moved toward the food source, a behavior absent in the fluoxetine-contaminated treatments. Other studies also observed fluoxetine’s effects on feeding behavior, such as food intake in the goldfish *Carassius auratus* at 54 μg/L^72^ or feeding rate in *H. diversicolor* at 10 μg/L.^9^ Changes in feeding behavior can directly affect population dynamics,^73^ making feeding-related endpoints highly ecologically relevant. In *L. variegatus*, feeding behavior is typically measured via egestion (i.e., the number of fecal pellets produced^74^). To our knowledge, we have described the first assay to test the worms’ ability to perceive food. The observed effects occurred after exposure to environmentally relevant concentrations (0.00025 mg/kg), indicating that fluoxetine may have detrimental effects in environments with limited food resources. However, all three behavioral assays were conducted during the recovery phase following fluoxetine exposure. Consequently, the observed behavioral responses may differ from those that would occur during continuous fluoxetine exposure.

Phototaxis is the responsive movement of an organism either toward or away from light.^75^ Changes in phototactic behavior can result from disruptions to phototactic sensors or changes in exploratory behavior (boldness). Neurotoxic compounds can disrupt phototaxis, impacting migration, settlement, and predator–prey dynamics.^76^ To our knowledge, this is the first assessment of chemical effects on phototactic behavior in *L. variegatus* since Monson et al.,^77^ who examined the combined effects of chemical and light exposure. Unexposed *L. variegatus* display a negative phototaxis by moving toward the shadow (a proxy for shelter). If this behavior is disrupted or even reversed, as shown in our study at environmentally relevant concentrations, it can have significant ecological consequences. Organisms with impaired negative phototactic behavior have a higher risk of predation, which is directly linked to survival and population-level effects.^76,78^

Oxidative stress and behavioral alterations, together with the assumption of higher energy maintenance costs, can be linked to effects on the conventional endpoints growth, reproduction, and mortality. In both chronic experiments, we observed reduced reproduction; however, the effect levels differed markedly despite similar reductions at the highest concentration. Subsequent research suggests that fluoxetine may delay development in *L. variegatus* rather than directly reduce reproduction (unpublished data). We also assume that the species undergoes two reproductive phases during the 28-day exposure, with the second occurring near the end of the experiment, based on the presence of recently fragmented worms lacking regenerated body parts. Therefore, biological variation between experiments is expected, especially if the chemical stressor leads to a delay in the development. This may explain the 100-fold difference in reproductive effect levels observed here and raises questions about whether the standard 28-day test adequately captures chronic effects in *L. variegatus*, particularly when substances can alter life cycle timing. We do not expect the observed differences in reproduction to translate into differences in the other ecotoxicological endpoints measured across the two chronic experiments, as we assume it is based on biological variation.

A range of effects was observed at intermediate, environmentally relevant concentrations, which diminished at higher concentrations, particularly for ETS activity, LPO levels, sediment avoidance, and growth. According to the NMDRC checkpoint criteria, no clear NMDRC could be identified, as the observed patterns were either driven by outliers or lacked sufficient effect strength. Nevertheless, several studies have reported NMDRCs following fluoxetine exposure, with pronounced effects at lower concentrations that diminish at higher concentrations.^79^ These patterns have been attributed to mechanisms such as receptor desensitization (i.e., reduced responsiveness following prolonged or repeated stimulation), the presence of multiple molecular targets with differing receptor affinities (as discussed above), or high-dose acute toxicity.^49^ Although a definitive NMDRC was not identified in this study, some endpoints suggest that *L. variegatus* may be particularly sensitive at intermediate fluoxetine concentrations. Further research, including more test concentrations in the relevant range, is needed to determine whether these responses reflect true NMDR relationships.

Our qualitative AOP demonstrates that exposure to the SSRI fluoxetine can cause molecular and adverse behavioral effects linked to the population level at environmentally relevant concentrations in the non-target organism *L. variegatus*, which is in line with previous studies reporting population-relevant fluoxetine effects.^26,80^ This could have significant impacts on the aquatic ecosystem, as it can disrupt *L. variegatus*’ function as a bioturbator^19^ and alter predator–prey relationships, for instance, with fish.^20^ Although the environmental impacts of pharmaceuticals have been demonstrated by numerous scientists over the years,^81,82^ they remain largely underregulated.^83,84^ Within the EU, the registration of chemicals is governed by well-established regulatory frameworks (e.g., Regulation 2006/121), which require ERAs as a prerequisite for market approval. However, human pharmaceuticals have historically faced less strict regulations due to ethical dilemmas. In 2024, the pharmaceutical ERA requirements were updated,^85^ but identifying a risk still does not prevent market authorization,^85^ and to date, no pharmaceutical has been denied market authorization due to ecological risks.^86^

The pharmaceutical ERA primarily relies on conventional endpoints (growth, reproduction, mortality).^85^ Reviewing experiments with the algae *Lemna* sp., the crustacean *Daphnia magna*, and the fish *Danio rerio* using only these conventional endpoints, Gunnarsson et al.^87^ derived a PNEC (predicted no-effect concentration) of 3.2 μg/L for fluoxetine, based on the lowest NOEC among the three taxonomic groups, which corresponds to a risk quotient (RQ; predicted environmental concentration/predicted no-effect concentration) of 0.4, which the authors interpreted as a moderate environmental risk.

However, many pharmaceutical effects manifest through alterations in neurotransmitters or hormones, which can induce behavioral changes, with consequences for survival and population dynamics.^88–90^ These NCEs are often far more sensitive than conventional endpoints.^17^ For *L. variegatus*, growth was significantly affected at 0.0025 mg/kg, reproduction at 0.25 mg/kg (Chronic I), and mortality (96 h LC50; Lethal Concentration 50%) at 81.4 mg/kg fluoxetine.^32^ With this, growth was 10 times, reproduction 1,000 times, and mortality 325,600 times less sensitive than the NCEs oxidative stress and behavior. When the NOECs (Table S3) are incorporated into the RQ calculation using environmental sediment concentrations detected in the EU (0.025 mg/kg in Spain; Castaño-Ortiz et al., 2024), the resulting RQ indicates a substantially higher risk, reaching 1, 10, and 100 when considering reproduction, chemical avoidance, and growth, respectively. For phototaxis and feeding, no NOEC could be determined, indicating that the risk is even higher. This raises questions about the conclusion that fluoxetine poses only a moderate environmental risk, particularly since the risk assessment did not consider sediment-dwelling species.^87^ If effects occur at concentrations that may fall below current analytical detection limits in sediment, such as in our study, this further complicates environmental risk characterization.

Behavioral endpoints seem especially sensitive indicators for the adverse effects of the neuroendocrine fluoxetine compared to conventional endpoints. Similar findings have been reported in other studies. For example, feeding and chemotactic behavior in *C. elegans* exposed to fluoxetine followed an NMDRC, with effects as low as 1 ng/L, far below the LC50 of 253 mg/L.^91^ Effects on social and predator-avoidance behavior in the fish *Pimephales promelas* occurred at 1 μg/L fluoxetine, 100 times more sensitive than reproductive effects (100 μg/L).^92^ The relevance of behavioral endpoints has now been acknowledged for the pharmaceutical ERA, particularly for neuroactive substances, and they are expected to be included in hazard assessment once standardized methods are available.^85^ We describe three novel behavioral assays that reveal ecologically relevant effects of pharmaceuticals on non-target organisms at environmental concentrations with important population-level implications that would go undetected under current regulatory frameworks. As also discussed in prior studies,^18,32^ we propose the inclusion of the three behavioral endpoints in OECD 225 to refine sediment hazard assessment.

## Supplementary Material



## Notes

Detailed description of the methodology for biochemical measurements for energy metabolism, neurotoxicity, oxidative stress and damage, IBR calculation, oxygen consumption, and AOP construction; Figures S1–S4; and Tables S1–S3 (PDF)
