# Supplementary material for: Digging into Understudied Sediments: What Lumbriculus variegatus Reveals about Fluoxetine Exposure from the Cellular to the Individual Level
Source: Environ Sci Technol. 2026 Sep 3;60(36):25192–205. doi: 10.1021/acs.est.5c12822 (PMC13585125; doi:10.1021/acs.est.5c12822)
Supplement: Supplementary file 1 [file es5c12822_si_001.pdf]

Supplementary information for 'Digging into Understudied Sediments: What *Lumbriculus variegatus* Reveals About Fluoxetine Exposure from the Cellular to the Individual Level.'

Journal: Environmental Science and Technology

Authors: Jacqueline Hilgendorf, Vânia Calisto, Diana Campos, Diogo N. Cardoso, Joana Fernandes, Diana L.D. Lima, Jesper G. Sørensen, Érika M.L. Sousa, Susana Loureiro*

Address: CESAM-Centre for Environmental and Marine Studies & Department of Biology, Campus Universitário de Santiago, University of Aveiro, 3810-193 Aveiro, Portugal.

Corresponding author (indicated by *): sloureiro@ua.pt

Summary: 15 pages; 4 Figures; 2 Tables

Table of Contents

***SI A1:** Biochemical analysis (for Section 2.3 in manuscript)* **3**

Energy metabolism 3

Neurotoxicity 4

Oxidative stress and damage 4

***SI A2:** Calculation of Integrated biomarker response (IBR) (for Section 2.3 in manuscript)* **4**

***SI A3:** Detailed description of oxygen consumption (for Section 2.4 in manuscript)* **5**

***SI A4:** AOP construction (for Section 3.5 in manuscript)* **5**

List of Figures

Figure S1: A: Electron transport system activity (ETS; mJ/h/mg organism); B: Sugar content (mJ/mg organism); C: Lipid content (mJ/mg organism); D: Protein content (mJ/mg organism); E: Cholinesterase activity (ChE, nmol/min/mg protein); F: lipid peroxidation activity (LPO, nmol TBARS/mg lipids); G: catalase activity (CAT, μmol/min/mg protein); H: glutathione-S-transferase activity (GST, nmol/min/mg protein); Level of I: total glutathione (TG, μM/mg protein), after chronic (28 d) exposure of Lumbriculus variegatus to the antidepressant Fluoxetine (6 concentrations ranging from 0.00025-25 mg/kg). All values are presented as mean ± SEM, n = 6. *denotes a significant ($p < 0.05$) difference when compared with the control (0) treatment following ANOVA and post-hoc Dunnett's tests........ 6

Table S1: Measurements of abiotic parameters (oxygen, conductivity, pH, and ammonia) during Chronic I. Initial measurements were conducted at the start of the experiment, and subsequent measurements were taken every 7 days. 7

Table S2: Measurements of abiotic parameters (oxygen, conductivity, pH, and ammonia) during Chronic II. Initial measurements were conducted at the start of the experiment, and subsequent measurements were taken every 7 days. 8

Figure S2: Oxygen consumption (μg oxygen/mg of organism/hour), after chronic (28 d) exposure of Lumbriculus variegatus to the antidepressant Fluoxetine (6 concentrations ranging from 0.00025-25 mg/kg). All values are presented as mean ± SEM, n = 3. 9

Figure S3:To obtain the integrated multi-biomarker response IBRv2, the absolute values calculated for each biomarker and each Fluoxetine treatment were summed (IBRv2= Σ|A|) for A: Biomarkers for oxidative stress and damage; and B: Biomarkers for energy metabolism. *denotes a significant ($p < 0.05$) difference when compared with the control (0) treatment following ANOVA and post-hoc Dunnett's tests. 9
Figure S4: Effects of chronic fluoxetine exposure on conventional endpoint reproduction of Lumbriculus variegatus. A: Reproduction of the organisms in the first chronic experiment. B: Reproduction of the organisms in the second chronic experiment. Reproduction is represented by the number of worms at the end of the experiment (mean ± SD) divided by adult worms (blue), offspring worms fully regrown (brown), and offspring worms recently fragmented (beige). 10

List of Tables

Table S1: Measurements of abiotic parameters (oxygen, conductivity, pH, and ammonia) during Chronic I. Initial measurements were conducted at the start of the experiment, and subsequent measurements were taken every 7 days. 7
Table S2: Measurements of abiotic parameters (oxygen, conductivity, pH, and ammonia) during Chronic II. Initial measurements were conducted at the start of the experiment, and subsequent measurements were taken every 7 days. 8
Table S3: NOECs (no observed effect concentrations) and LOECs (lowest observed effect concentrations) for all measured endpoints in Lumbriculus variegatus following fluoxetine exposure in this study. No effect: no effect was observed at any tested fluoxetine concentration. N.d.: an effect was observed at the lowest tested concentration; therefore, a NOEC could not be determined. 10

SI A1: Biochemical analysis (for Section 2.3 in manuscript)

Before analysis, the samples destined to evaluate oxidative stress and damage and neurotoxicity (A) were homogenised in 1600 µL of 0.1M K-phosphate buffer (pH 7.4), while the samples for energy metabolism (B) were homogenised in 1600 mL of Milli-Q water, both using a Tissuelyser II Qiagen (40 sec, 30 Hz). From samples A, a 200 µl aliquot was allocated for LPO analysis, with 4 µL of 4% BHT (2,6-Di-tert-butyl-4-methylphenol) in methanol added before processing. The remaining homogenate was centrifuged at 10000 g for 20 minutes at 4 °C to separate the post-mitochondrial supernatant (PMS). The PMS was then aliquoted into microtubes and stored at −80 °C for subsequent analysis of catalase activity (CAT), glutathione-*S*-transferase activity (GST), total glutathione (TG) levels, and protein quantification. Two aliquots were taken from each sample B, one for energy reserves analysis, including lipid, sugar, and protein content, while another aliquot was used to assess aerobic energy production by estimating electron transport system (ETS) activity.

Energy metabolism

Total lipid content (300µL) was extracted with chloroform and methanol. The organic phase was collected and then acidified with sulfuric acid, and absorbance was measured at 375 nm, and tripalmitin was used as a standard for quantification. For the sugar and protein content (300 µL), proteins were precipitated with trichloroacetic acid in another aliquot, followed by incubation at −20 °C. The sugar content was determined in the supernatant by incubating the samples with phenol and sulfuric acid, and the absorbance was measured at 492 nm, using glucose as a standard for quantification. The pellets from this second aliquot were resuspended in sodium hydroxide and incubated at 60 °C for 30 min to measure the protein content. Protein content was quantified using the Bradford method at 592 nm (Bradford, 1976), using bovine serum albumin as the standard for quantification. Obtained values were converted into caloric values using the enthalpy of combustion: 39.5 kJ/g lipid, 17.5 kJ/g glycogen, and 24 kJ/g protein. Lipids, sugars, and proteins were expressed as mJ/mg organism.

The ETS aliquot (300µL) was added to the homogenisation buffer and then centrifuged at 1000g for 10 min at 4°C. ETS activity was assessed in supernatant by incubating samples with a reaction solution containing nicotinamide-adenine dinucleotide (NADH), nicotinamide-adenine dinucleotide phosphate (NADPH), and Iodonitrotetrazolium chloride (INT), with absorbance measured at 490 nm over 3 minutes. ETS activity was converted into caloric values using specific oxyenthalpic equivalents (480 kJ/mol O_2) and expressed as mJ/h/mg organism.

Neurotoxicity

In a study by Chuiko et al. (2011, measuring cholinesterase in *L. variegatus*, the worms did not display any substrate specificity. The authors concluded that only atypical cholinesterase (ChE) is present in the worm's tissues, which differs from the typical acetyl- and butyrylcholinesterases found in mammals. Therefore, the enzyme is described as ChE in this study. However, a slightly higher activity was observed in measurements with acetylthiocholine (ACh) as substrate (Chuiko et al., 2011). Based on this, ChE activity was measured in 50 μL of PMS using Ellman's method (Ellman et al., 1961), with ACh as the substrate, and absorbance was recorded at 412 nm (Guilhermino et al., 1996). ChE activity was expressed as nmol/min/mg protein.

Oxidative stress and damage

LPO was assessed in 200μL of homogenised samples by measuring thiobarbituric acid-reactive substances (TBARS) at 535 nm, following the method of Bird & Draper (1984). LPO was expressed as nmol TBARS/mg protein. GST activity was determined in 50μL of PMS using the colourimetric conjugation reaction between Glutathione (GSH) and 1-chloro-2,4-dinitrobenzene (DNCB), measured at 340 nm (Habig et al., 1974). GST activity was expressed as nmol/min/mg protein. CAT activity was quantified in 10 μL of PMS by measuring the decomposition of H_2O_2 at 240 nm (Clairborne, 1989). CAT activity was expressed as μmol/min/mg protein.

TG levels were measured in 50 μL of PMS following the recycling reaction of reduced glutathione in excess of glutathione reductase by adding DTNB (Ellman's Reagent - 5,5-dithio-bis-(2-nitrobenzoic acid), and NADPH, with absorbance measured at 412 nm for 3 minutes (Baker et al., 1990). TG was expressed as μM/ mg protein, and L-GSH was used as a standard.

The protein concentration was measured following the Bradford method, using bovine γ-globulin as a standard (Bradford, 1976).

SI A2: Calculation of Integrated biomarker response (IBRv2) (for Section 2.3 in manuscript)

The integrated biomarker response (IBR) combines all analysed biomarkers into a single response that is visualised in a star plot. We calculated the revised version of the IBR (IBRv2) as described by Sanchez et al. (2013). The mean values of the biomarker deviation index A were computed and reported in a star plot (Figure 3) to represent the reference deviation of each investigated biomarker. For the IBRv2 calculation, a log transformation was applied to the individual biomarker data (X_i) to reduce variance: $Y_i = \log(X_i)$. The general mean (μ) and standard deviation (s) of Y_i were computed, and Y_i was standardised: $Z_i = (Y_i - \mu/s)$. The next step was to create a baseline centred on zero and to represent biomarker variation according

to this baseline. For this, the mean of standardised biomarker response (Z_i) and the mean of reference biomarker data (Z_0) were used to define a biomarker deviation index (A): $A = Z_i - Z_0$. To obtain the integrated multi-biomarker response IBRv2, the absolute values of A calculated for each biomarker and each Fluoxetine treatment were summed: $IBRv2 = \Sigma|A|$. Biochemical endpoints were grouped into two main categories: 1) markers of energy metabolism (protein, lipids, sugar, and ETS activity), and 2) markers of oxidative stress (TG, GST, CAT), oxidative damage (LPO), and neurotoxicity (ChE). The area from 1 to 0 reflects biomarker induction, and the area below 0 indicates biomarker inhibition.

SI A3: Detailed description of oxygen consumption (for Section 2.4 in manuscript)

Syringes were filled with 50 mL of ASTM water and the organisms, ensuring all air was expelled, and were then incubated in the dark in a water bath maintained at 20 °C. After 2 hours, initial oxygen concentrations were recorded using an oxygen meter (model 782, Strathkelvin Instruments, Glasgow) paired with an oxygen electrode (model 1302, Strathkelvin Instruments, Glasgow). Samples were collected using a 0.5 mL gas-tight syringe and manually injected into the electrode chamber (volume = 70 µL) at a constant rate of 0.5 mL min^{-1}, with readings taken after 1 minute. Final oxygen concentrations were measured in the same manner after 22 additional hours.

At the end of the 24-hour exposure period, worms were removed from the syringes, dried at 60 °C for 2 days, and weighed. Oxygen consumption was calculated as the difference in oxygen content between the initial (T_0 = 2 h) and final (T_{final} = 24 h) time points. Respiration rates were expressed as µg oxygen consumed/mg of organism/hour.

SI A4: AOP construction (for Section 3.5 in manuscript)

A qualitative AOP was developed based on the OECD AOP framework (OECD, 2017), integrating the measured endpoints on all biological levels. To better understand the effects of chemicals, it is essential to identify the molecular initiating event (MIE), assess key events (KEs) at both the molecular and cellular levels, and causally link them to adverse outcomes (AOs) at both the individual and population levels (ECHA, 2024b). The most effective way to establish this connection is through the conceptual framework of an AOP (Ankley et al., 2010; OECD, 2017). Only endpoints showing significant treatment-related effects were included in the pathway. The resulting AOP diagram offers mechanistic insight into the MoA of Fluoxetine and the linkage of cellular effects to the population level. Integrating all results supports the interpretation of observed toxic effects within a regulatory context.

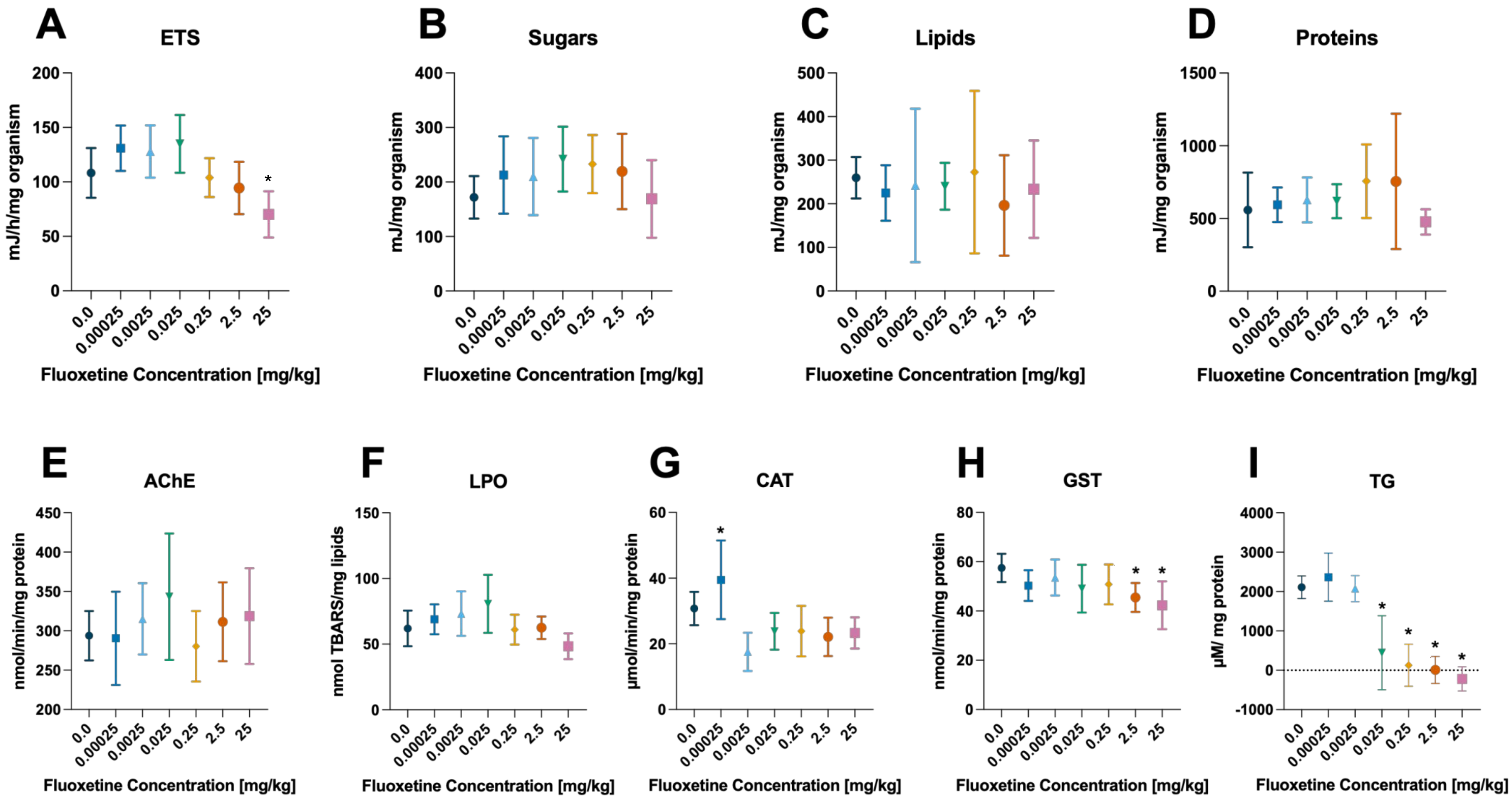

Figure S1: *A: Electron transport system activity (ETS; mJ/h/mg organism); B: Sugar content (mJ/mg organism); C: Lipid content (mJ/mg organism); D: Protein content (mJ/mg organism); E: Cholinesterase activity (ChE, nmol/min/mg protein); F: lipid peroxidation activity (LPO, nmol TBARS/mg lipids); G: catalase activity (CAT, µmol/min/mg protein); H: glutathione-S-transferase activity (GST, nmol/min/mg protein); Level of I: total glutathione (TG, µM/mg protein), after chronic (28 d) exposure of Lumbriculus variegatus to the antidepressant Fluoxetine (6 concentrations ranging from 0.00025-25 mg/kg). All values are presented as mean ± SEM, n = 6. *denotes a significant ($p < 0.05$) difference when compared with the control (0) treatment following ANOVA and post-hoc Dunnett's tests.*

Table S1: *Measurements of abiotic parameters (oxygen, conductivity, pH, and ammonia) during Chronic I. Initial measurements were conducted at the start of the experiment, and subsequent measurements were taken every 7 days.*

Replicate	O2 [mg/l]					Conductivity [mS]					pH					Ammonia [mg/L]				
	T0	T7	T14	T21	T28	T0	T7	T14	T21	T28	T0	T7	T14	T21	T28	T0	T7	T14	T21	T28
CT1	7.2	6.1	6.0	5.5	4.9	0.908	1.106	0.913	0.932	0.879	7.56	8.43	8.4	8.79	8.63	0.25	0.25	0.5	0.5	0.5
CT2	7.1	5.3	7.3	7.5	5.7	0.899	1.034	0.917	0.916	0.874	8.02	8.50	8.64	9.06	8.63					
CT3	6.5	6.2	7.1	6.4	6.9	0.954	0.945	0.842	0.971	0.789	7.55	8.45	8.54	8.95	8.53					
T1.1	5.7	4.9	5.5	5.2	5.5	0.934	0.940	0.940	0.940	0.809	7.89	8.43	8.43	8.78	8.55	0.25	0.25	0.25	0.5	0.5
T1.2	6.3	5.7	4.6	5.0	5.2	0.978	0.962	0.986	0.939	0.812	8.02	8.43	8.19	8.79	8.61					
T1.3	6.9	6.3	6.8	4.3	4.6	0.956	0.931	0.928	0.969	0.853	7.98	8.44	8.55	8.66	8.43					
T2.1	5.6	5.1	5.5	5.7	5.4	0.877	0.933	0.950	0.948	0.821	8.05	8.46	8.42	8.89	8.47	0.25	0.25	0.25	0.5	0.5
T2.2	6.7	4.6	4.6	4.6	5.2	0.899	0.904	0.933	0.936	0.796	8.11	8.47	8.31	8.66	8.47					
T2.3	7.1	5.2	7.0	6.8	6.7	0.911	0.931	0.918	0.927	0.794	8.23	8.40	8.6	8.90	8.70					
T3.1	6.6	4.0	6.0	6.4	6.4	0.965	0.978	0.966	0.953	0.825	7.98	8.34	8.51	8.93	8.65	0.25	0.25	0.5	0.5	1.5
T3.2	6.4	5.9	5.1	5.1	5.2	0.966	0.940	0.951	0.973	0.836	7.86	8.52	8.39	8.72	8.43					
T3.3	6.8	4.9	6.1	7.1	6.8	0.876	0.956	0.951	0.925	0.779	8.20	8.32	8.49	9.00	8.75					
T4.1	5.6	5.2	6.8	6.8	6.4	0.834	0.929	0.930	0.921	0.817	8.32	8.55	8.53	8.98	8.64	0.25	0.25	0.25	0.5	1.0
T4.2	7.1	5.2	6.8	6.6	7.3	0.978	0.956	0.946	0.932	0.793	7.84	8.55	8.49	8.86	8.83					
T4.3	5.6	4.2	6.0	5.4	5.7	0.988	0.949	0.936	0.938	0.806	7.93	8.29	8.6	8.74	8.60					
T5.1	6.7	5.0	5.6	4.5	5.4	0.865	0.994	0.973	0.978	0.816	8.02	8.42	8.36	8.65	8.59	0.25	0.25	0.25	0.5	1.5
T5.2	6.7	7.6	6.3	6.7	7.0	0.856	0.958	0.935	0.934	0.803	8.05	8.37	8.56	8.84	8.73					
T5.3	7.2	5.0	6.2	5.1	5.6	0.933	0.936	0.922	0.933	0.851	8.13	8.39	8.47	8.59	8.52					
T6.1	6.8	5.4	7.2	6.3	6.6	0.923	0.953	0.921	0.908	0.796	8.23	8.40	8.59	8.8	8.69	0.25	0.25	0.25	0.5	1.5
T6.2	6.9	4.6	6.8	4.7	5.6	0.945	0.940	0.899	0.923	0.824	7.67	8.46	8.52	8.73	8.48					
T6.3	7	5.0	7.2	5.4	7.0	0.987	0.962	0.931	0.925	0.865	7.98	8.38	8.67	8.87	8.65					

Table S2: *Measurements of abiotic parameters (oxygen, conductivity, pH, and ammonia) during Chronic II. Initial measurements were conducted at the start of the experiment, and subsequent measurements were taken every 7 days.*

Replicate	O2 [mg/l]					Conductivity [mS]					pH					Ammonia [mg/L]				
	T0	T7	T14	T21	T28	T0	T7	T14	T21	T28	T0	T7	T14	T21	T28	T0	T7	T14	T21	T28
CT1	6.3	5.7	7.2	7.8	7.0	0.789	0.866	0.880	0.876	0.833	7.55	8.55	8.47	8.59	8.78	<0.25	<0.25	0.25	0.25	0.5
CT2	6.0	6.1	7.2	6.9	6.9	0.769	0.836	0.877	0.855	0.775	7.55	8.56	8.49	8.45	8.70					
CT3	5.9	6.1	7.0	6.8	7.1	0.767	0.827	0.872	0.862	0.810	7.53	8.56	8.51	8.50	8.73					
T1.1	6.4	6.0	7.6	6.8	8.6	0.778	0.888	0.876	0.873	0.846	7.62	8.50	8.52	8.57	8.83	<0.50	<0.25	0.25-0.5	0.5	0.5
T1.2	5.2	6.3	6.7	6.9	7.1	0.750	0.883	0.858	0.860	0.796	7.40	8.53	8.52	8.47	8.80					
T1.3	6.4	6.3	6.6	7.0	7.3	0.770	0.877	0.854	0.852	0.803	7.70	8.55	8.50	8.55	8.70					
T2.1	5.9	6.0	7.2	7.2	8.4	0.769	0.883	0.882	0.837	0.840	7.60	8.27	8.49	8.52	8.80	<0.25	<0.25	0.5	0.5	0.5
T2.2	6.5	6.1	7.0	7.1	7.4	0.777	0.861	0.837	0.852	0.797	7.72	8.55	8.42	8.49	8.75					
T2.3	6.5	6.1	6.8	7.7	7.5	0.785	0.863	0.877	0.862	0.816	7.70	8.48	8.44	8.45	8.79					
T3.1	6.1	3.7	7.0	6.6	7.6	0.791	0.879	0.869	0.875	0.870	7.56	8.12	8.53	8.32	8.77	<0.25	<0.50	0.5	0.5	0.5
T3.2	5.2	5.5	6.8	7.5	7.3	0.747	0.847	0.871	0.882	0.836	7.27	8.45	8.51	8.37	8.70					
T3.3	5.4	5.0	6.5	6.5	6.9	0.850	0.867	0.878	0.847	0.807	7.27	8.51	8.49	8.38	8.61					
T4.1	7.0	6.8	7.7	6.9	6.9	0.734	0.842	0.925	0.913	0.835	7.14	8.44	8.55	8.47	8.77	<0.25	<0.50	0.5	0.5	0.5
T4.2	5.4	6.4	6.9	6.8	6.5	0.753	0.842	0.886	0.862	0.801	7.40	8.48	8.51	8.38	8.70					
T4.3	4.6	6.3	6.8	7.0	7	0.733	0.865	0.869	0.871	0.796	7.21	8.46	8.53	8.53	8.80					
T5.1	5.2	5.4	6.9	6.8	5.9	0.755	0.821	0.859	0.843	0.932	7.05	8.11	8.56	8.21	8.31	<0.25	<0.50	0.5	0.5	0.5
T5.2	5.3	4.7	7.1	7	5.2	0.799	0.845	0.844	0.869	0.856	7.22	8.23	8.34	8.53	8.12					
T5.3	4.9	6.8	5.6	5.9	6.7	0.788	0.786	0.799	0.846	0.827	7.13	8.22	8.63	8.12	8.24					
T6.1	5.4	7.0	5.8	7.1	4.9	0.766	0.756	0.845	0.911	0.921	6.99	8.13	8.32	8.31	8.29	<0.25	<0.50	0.5	0.5	0.5
T6.2	5.8	5.6	6.1	6.4	5.4	0.793	0.866	0.842	0.843	0.873	7.43	8.45	8.12	8.42	8.41					
T6.3	7.1	4.9	6.2	6.8	5.9	0.755	0.856	0.822	0.954	0.822	7.12	8.34	8.43	8.21	8.11					

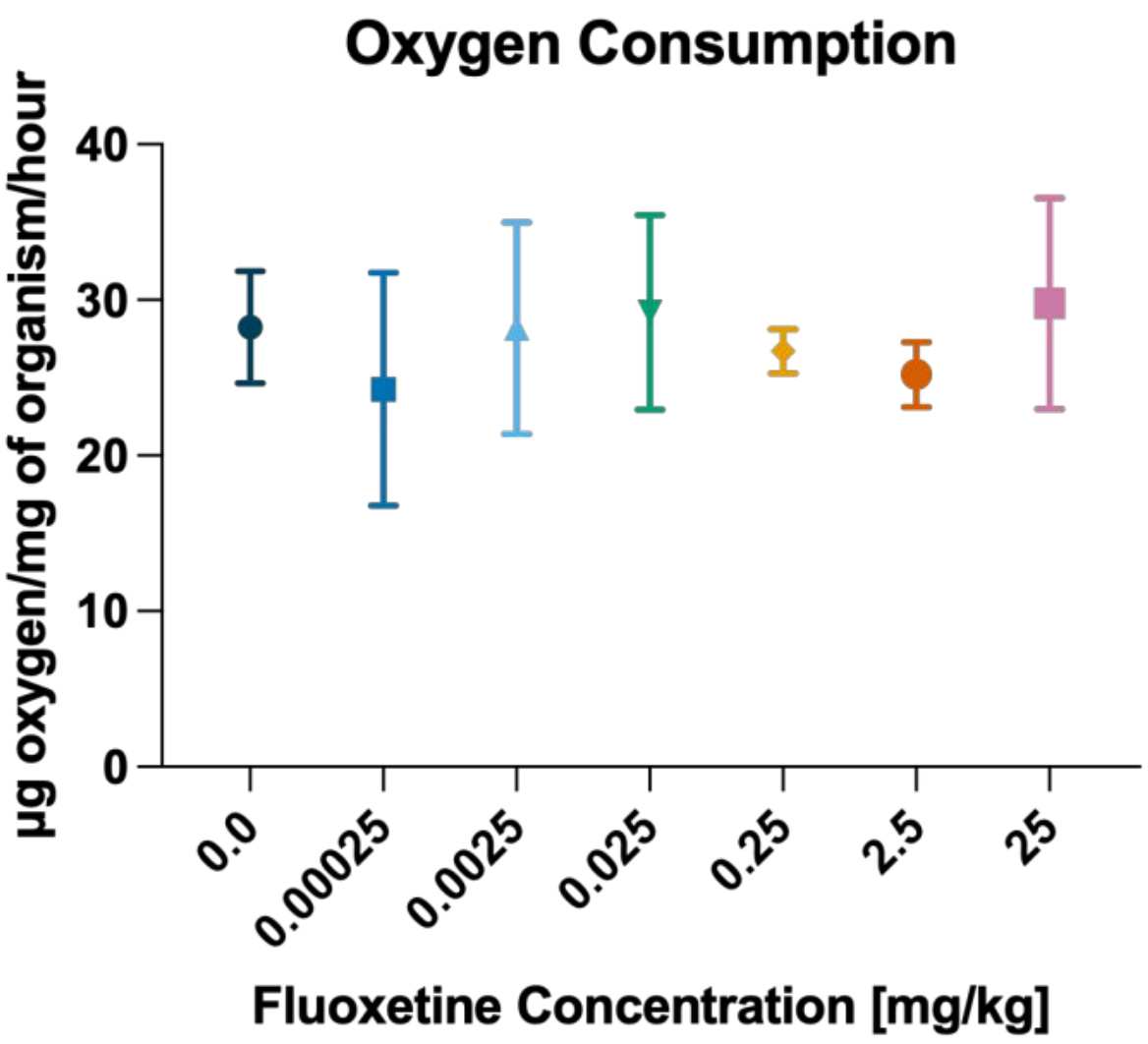

Figure S2: *Oxygen consumption (µg oxygen/mg of organism/hour), after chronic (28 d) exposure of Lumbriculus variegatus to the antidepressant Fluoxetine (6 concentrations ranging from 0.00025-25 mg/kg). All values are presented as mean ± SEM, n = 3.*

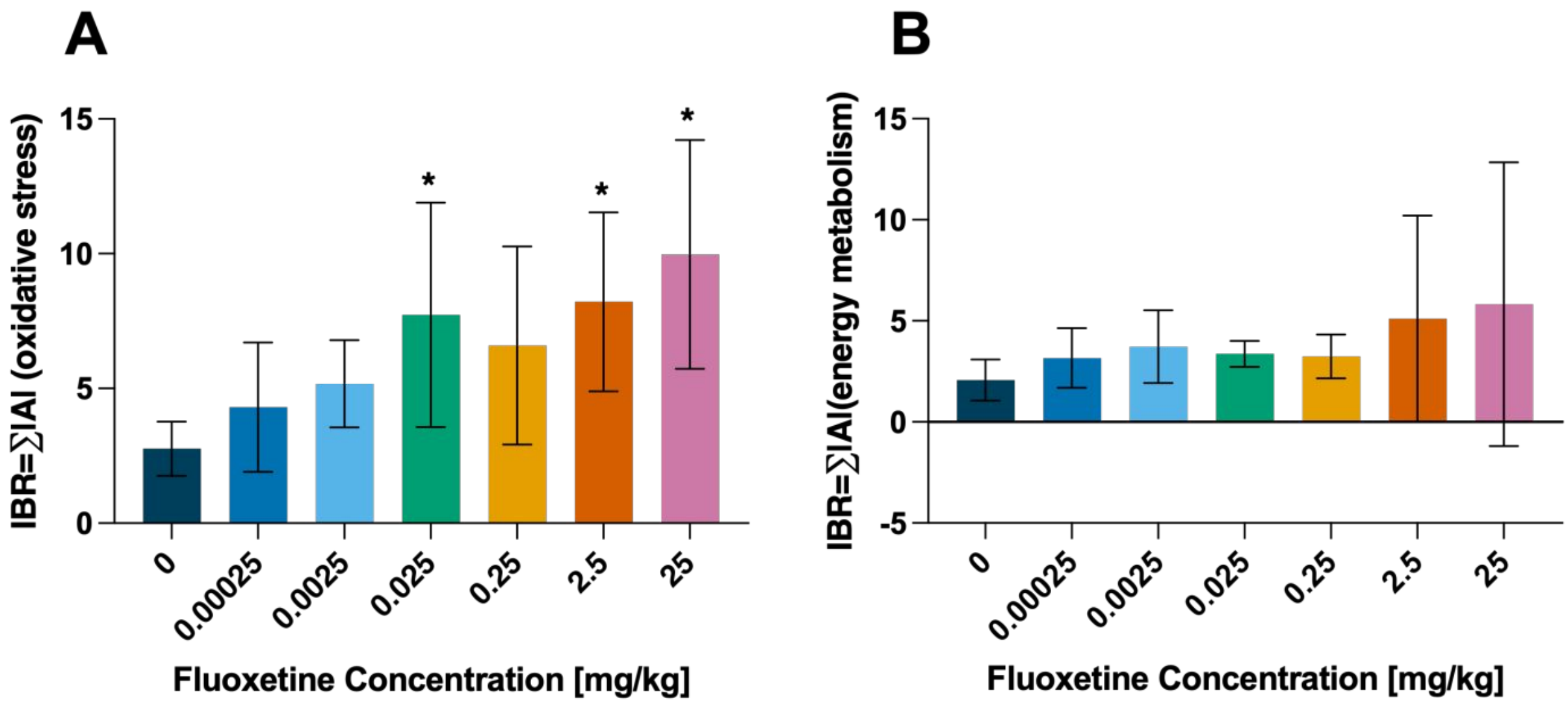

Figure S3*: To obtain the integrated multi-biomarker response IBRv2, the absolute values calculated for each biomarker and each Fluoxetine treatment were summed (IBRv2= Σ|A|) for A: Biomarkers for oxidative stress and damage; and B: Biomarkers for energy metabolism. *denotes a significant (p < 0.05) difference when compared with the control (0) treatment following ANOVA and post-hoc Dunnett's tests.*

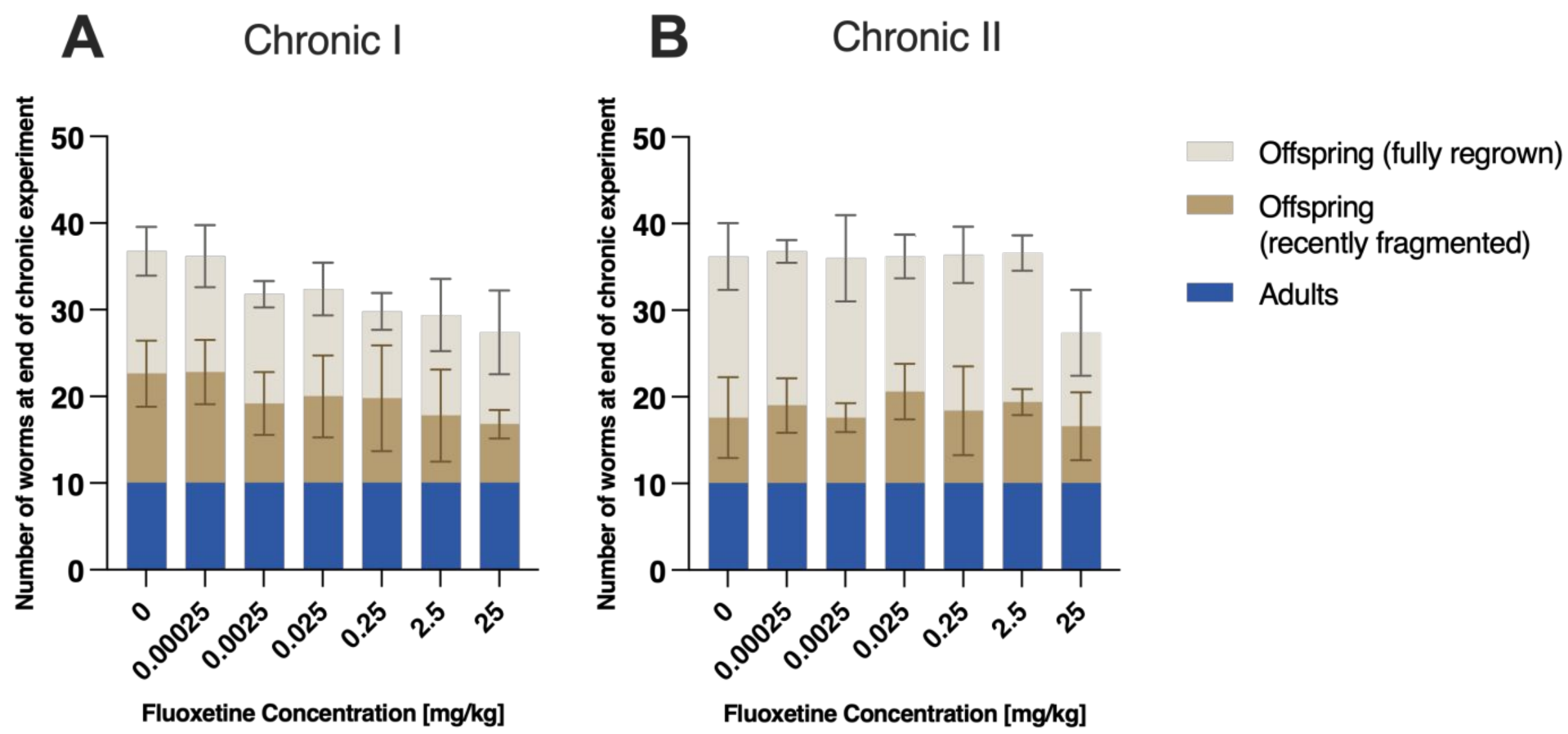

***Figure S4**: Effects of chronic fluoxetine exposure on conventional endpoint reproduction of Lumbriculus variegatus. A: Reproduction of the organisms in the first chronic experiment. B: Reproduction of the organisms in the second chronic experiment. Reproduction is represented by the number of worms at the end of the experiment (mean ± SD; n control = 10; n exposure treatments = 5) divided by adult worms (blue), offspring worms fully regrown (brown), and offspring worms recently fragmented (beige).*

***Table S3:** NOECs (no observed effect concentrations) and LOECs (lowest observed effect concentrations) for all measured endpoints in Lumbriculus variegatus following fluoxetine exposure in this study. No effect: no effect was observed at any tested fluoxetine concentration. N.d.: an effect was observed at the lowest tested concentration; therefore, a NOEC could not be determined.*

Endpoint	NOEC [mg/kg]	LOEC [mg/kg]
Cholinesterase	no effect	no effect
Sugar	no effect	no effect
Protein	no effect	no effect
Carbohydrates	no effect	no effect
Electron Transport System	2.5	25
Total Glutathione	0.025	0.025
Glutathione-S-transferase	0.25	2.5
Catalase	n.d.	0.00025
Lipid Peroxidation	2.5	25
Oxygen consumption	no effect	no effect
Phototactic behaviour	n.d.	0.00025
Chemical avoidance behaviour	0.0025	0.025
Feeding behaviour	n.d.	0.00025
Reproduction	0.025	0.25
Growth	0.00025	0.0025
